# Human-like cutaneous neuropathologies associated with a porcine model of peripheral neuritis: A translational platform for neuropathic pain

**DOI:** 10.1016/j.ynpai.2018.07.002

**Published:** 2018-07-20

**Authors:** Frank L. Rice, David Castel, Elizabeth Ruggiero, Marilyn Dockum, George Houk, Itai Sabbag, Phillip J. Albrecht, Sigal Meilin

**Affiliations:** a Neuroscience & Pain Research Group, Integrated Tissue Dynamics, LLC, Rensselaer, NY 12144, United States; b Division of Health Sciences, University at Albany, Rensselaer, NY 12144, United States; c The Neufeld Cardiac Research Institute, Sheba Medical Centre, Sackler School of Medicine, Tel-Aviv University, Tel-Aviv 69978, Israel; d Lahav Research Institute, Kibutz Lahav, Negev 85335, Israel; e MD Biosciences, Neurology R&D Division, Nes-Ziona 74140, Israel

**Keywords:** Animal model, IENF density, PGP9.5, CGRP, ET-1 receptors, NaV, Immunolabeling

## Abstract

Despite enormous investment in research and development of novel treatments, there remains a lack of predictable, effective, and safe therapeutics for human chronic neuropathic pain (NP) afflictions. NP continues to increase among the population and treatments remain a major unmet public health care need. In recent years, numerous costly (time and money) failures have occurred attempting to translate successful animal pain model results, typically using rodents, to human clinical trials. These continued failures point to the essential need for better animal models of human pain conditions. To address this challenge, we have previously developed a peripheral neuritis trauma (PNT) model of chronic pain induced by a proximal sciatic nerve irritation in pigs, which have a body size, metabolism, skin structure, and cutaneous innervation more similar to humans. Here, we set out to determine the extent that the PNT model presents with cutaneous neuropathologies consistent with those associated with human chronic NP afflictions. Exactly as is performed in human skin biopsies, extensive quantitative multi-molecular immunofluorescence analyses of porcine skin biopsies were performed to assess cutaneous innervation and skin structure. ChemoMorphometric Analysis (CMA) results demonstrated a significant reduction in small caliber intraepidermal nerve fiber (IENF) innervation, altered dermal vascular innervation, and aberrant analgesic/algesic neurochemical properties among epidermal keratinocytes, which are implicated in modulating sensory innervation. These comprehensive pathologic changes very closely resemble those observed from CMA of human skin biopsies collected from NP afflictions. The results indicate that the porcine PNT model is more appropriate for translational NP research compared with commonly utilized rodent models. Because the PNT model creates cutaneous innervation and keratinocyte immunolabeling alterations consistent with human NP conditions, use of this animal model for NP testing and treatment response characteristics will likely provide more realistic results to direct successful translation to humans.

## Highlights

• Pig peripheral neuritis trauma model mimics cutaneous human pathologies.
• Pig skin anatomically similar to humans, unlike rodent.
• Pig PNT model provides better data than rodent for translational pain research.

## 1 Introduction

Neuropathic pain (NP) remains a clinical burden and treatment challenge. The lack of safe and effective treatments for debilitating NP associated with a wide variety of peripheral neuropathies, including various forms of neuritis caused by inflammation, continues to be one of the foremost health care challenges, despite an enormous investment in research (Chaplan et al., 2010, Borsook et al., 2014, Javed et al., 2015, Singla et al., 2015, Bruehl et al., 2016, Dworkin et al., 2016, Maixner et al., 2016, Colloca et al., 2017, Dworkin and Edwards, 2017). Among these afflictions are neuropathies associated with various types of nerve traumas ranging from overt transections and crushes (e.g., motor vehicle accidents), to insidious compression, irritation, and inflammation (e.g., sciatica), to inexplicably minor soft tissue damage (e.g., Complex Regional Pain Syndrome type I), and metabolic disorders (e.g., diabetes), among numerous other causes. Current NP therapeutics typically provide limited, unpredictable relief with a high incidence of deleterious side effects, while promising therapeutics, primarily developed and tested in rodent animal models, have repeatedly failed to translate to success in human NP clinical trials. Rodent models of traumatic nerve injury are commonly used for NP research because of their relative simplicity, reproducibility, and low cost (Wang and Wang, 2003). The most widely used and accepted models include sciatic nerve crush (Varejao et al., 2004, Nichols et al., 2005, Baptista et al., 2008), partial sciatic nerve ligation (Seltzer et al., 1990), spinal nerve ligation (Kim and Chung, 1992), spared nerve injury (Decosterd and Woolf, 2000, Pertin et al., 2012), and loose-ligature suture irritation referred to as chronic constriction injury (CCI) (Bennett and Xie, 1988). Of these rodent models, CCI is among the least invasive, yet causes robust pain behaviors and is perhaps most representative of human peripheral neuritis due to nerve irritation, inflammation, and constriction. However, the continued failure of pain clinical trials indicates an essential need for animal models that better represent the human disease conditions for testing and data generation that will better direct successful translation to humans.

To address the challenge of translating experimentally induced pain in animal models to successful human applications (Hill, 2000, Rice et al., 2008, Borsook et al., 2014, Hama and Takamatsu, 2016, Shidahara et al., 2016), we have developed a modified CCI peripheral neuritis trauma (PNT) model in pigs which, unlike rodents, have a close anatomical, physiological, and neurological similarity to humans (Rukwied et al., 2008, Schley et al., 2012, Swindle et al., 2012, Hirth et al., 2013, Castel et al., 2016). In particular, the structure and innervation of pig skin is especially more like that of humans (Rukwied et al., 2008, Dusch et al., 2009, Janczak et al., 2012, Schley et al., 2012, Di Giminiani et al., 2013, Hirth et al., 2013), providing a platform for comprehensive ChemoMorphometric Analysis (CMA) of skin biopsies which have been increasingly used to discover pathologies associated with a variety of painful peripheral neuropathies in humans (Holland et al., 1997, Kennedy and Wendelschafer-Crabb, 1999, Polydefkis et al., 2001, Obermann et al., 2008, Sommer, 2008, Vlckova-Moravcova et al., 2008, Lauria et al., 2009, Weis et al., 2011, Hoeijmakers et al., 2012, Boyette-Davis et al., 2013, Cheng et al., 2013, Uceyler et al., 2013, Grone et al., 2014, Doppler et al., 2015, Hoeijmakers et al., 2015, Divisova et al., 2016). As a large animal model, pigs also present as a more cost-effective and ethically acceptable alternative to non-human primate use for NP research.

Two particularly important discoveries have been made using skin biopsies from humans with a variety of painful peripheral neuropathies. First, a reduction is often found among small caliber unmyelinated and lightly myelinated innervation (C and Aδ fibers, respectively), particularly from the epidermis, which are a presumed source of NP. Although a reduction in the innervation that is implicated in pain reception seems paradoxical to the generation of increased pain, electrophysiological assessments indicate that remaining small caliber innervation becomes hyperactive. The innervation hyperactivity has mostly been attributed to pathologically altered properties among the primary sensory neurons located in the dorsal root ganglia (Serra, 1999, Ochoa et al., 2005, Kleggetveit et al., 2012, Hirth et al., 2013, Serra et al., 2014). However, a second factor discovered in human biopsies that most likely contributes to innervation hyperexcitability involves the layers of epidermal keratinocytes which make up the majority of the target field for small-fiber epidermal innervation. It has now been demonstrated that keratinocytes normally express a differentially stratified distribution of excitatory and inhibitory neural signaling systems that are implicated in modulation of sensory ending activity, and appear skewed towards an excitatory imbalance among several human NP afflictions (Khodorova et al., 2003, Ibrahim et al., 2005, Dussor et al., 2009, Barr et al., 2013, Baumbauer et al., 2015, Pang et al., 2015). Additionally, this excitatory imbalance among epidermal keratinocytes can be induced in rodent experimental pain models involving proximal sciatic nerve traumas, indicative of a cross-talk between the innervation and keratinocytes (Hou et al., 2011). The purpose of this study was to further validate the pig sciatic PNT model by using multi-molecular immunofluorescence assessments (CMA) of skin biopsies to profile the impact on cutaneous innervation and epidermal keratinocytes relative to those observed in human NP afflictions.

## 2 Methods

### 2.1 Overall study design

A total of 38 male Danish Landrace × Large White crossbred pigs (*Sus domestica*) from the domestic herd at Lahav Laboratories, Negev, Israel, were used in this study (Table 1). All pigs were 9-weeks old, weaned, and weighed 15 ± 1 kg at initiation. Prior to study initiation, all pigs were kept under conventional production conditions. Pigs were housed in open pens (1.4 × 2.4 m) in groups of 2–3 on a 12 hr light-dark cycle for the 7 days prior to study initiation. Feeding occurred three times daily using specific pig food (Dry Sows; Ct # 5420; Milobar, 7880, Oshrat, Israel), and pigs were provided opportunities to root and chew for enrichment. Fresh water was provided *ad libitum* by an automated system.

**Table 1:** Pig preparations.

Part 1 Lesion Impact	Post-Operative Day of Euthanasia					
*Ipsilateral Biopsy Only*	POD 10		POD 18		POD 28	
	Group	N	Group	N	Group	N
Full Nerve Crush (FC)			FC18	4		
Partial Nerve Crush (PC)	PC10	3			PC28	4
Peripheral Neuritis Trauma (PNT)					PNT28	8
Sham					Sham28	3

Part 2 Impact Time-course	Post-Operative Day of Euthanasia							
*Ipsilateral & Contralateral Biopsy*	POD 1		POD 7		POD 14		POD 21	
	Group	N	Group	N	Group	N	Group	N
Peripheral Neuritis Trauma (PNT)	PNT1	4	PNT7	4	PNT14	4	PNT21	4

The animal procedures consisted of four phases: (1) habituation (Pre-Op Day −5 to −1); (2) surgery (Day 0); (3) follow-up on Post-Operative Days (POD) ranging from 1 to 28; and (4) euthanasia and skin biopsy ranging from POD1 to 28 (see below). All procedures and experiments were approved by the M.D. Biosciences Institutional Animal Care and Use Committee (IACUC) with adherence to National Institutes of Health guidelines, and were designed to reduce numbers and undue suffering in accordance with the IASP (International Association for the Study of Pain) (Zimmermann, 1983). At the conclusion of all experiments, pigs were humanely euthanized according to animal welfare guidelines on the POD shown in Table 1:

The study consisted of two parts (Table 1):

1. *Impact of sciatic nerve lesions.* The focus of this study was on a peripheral neuritis trauma (PNT) model in which sustained robust pain behaviors were induced by loose ligatures, presoaked in Complete Freund Adjuvant, tied around the most proximal portion of the sciatic nerve. Following up to a four week assessment of pain behaviors, multi-molecular immunolabeling CMA of skin biopsies from the pain afflicted areas were performed to investigate PNT impact on cutaneous innervation and stratified epidermal keratinocyte neuromodulator properties. Additional data from comparable assessments are included from some pigs involving full crushes (FC) and partial crushes (PC) of the proximal sciatic nerve, although for ethical and/or technical reasons these animals were not maintained beyond POD18.
2. *Time course of PNT-induced pathologies.* Having defined the pathologies associated with PNT insult after 4 weeks, a second set of experiments was conducted to assess the onset and time course of the PNT-induced pathologies.

### 2.2 Nerve interventions

#### 2.2.1 Habituation

Pigs were habituated to the study protocol for 5 days prior to surgery, as described previously (Castel et al., 2014, Castel et al., 2016). The habituation process was conducted to reduce novel stress associated with the experiments, including familiarizing pigs with the study schedule and technicians. The pigs were trained to walk to the preparation room daily during the habituation period and were always returned to their original pens with their original pen mates. The body weight of the pigs was measured at the beginning of the acclimatization period (five days prior to surgery), on the day of surgery prior to anesthesia, and thereafter once weekly post-surgery until the study end. The temperature in the surgery room was maintained at 19 °C (range 18–20 °C).

#### 2.2.2 Anesthesia and surgery

On the day of surgery (Day 0), each pig walked freely to the preparation room. The pigs were anesthetized by inhalation of 3% isoflurane/100% oxygen mixture using a face mask, and the entire duration of the surgery procedures was approximately 30 min. Preparation for the various models and detailed surgical methods have been previously described (Castel et al., 2014, Castel et al., 2016). The proximal portion of the sciatic nerve was exposed for the following procedures (Table 1):

1. *Full Nerve Crush (FC) [n = 4]:* A hemostat was clamped across the entire width of the sciatic nerve for a period of 30 s to create a 10 mm long crush injury.
2. *Partial Nerve Crush (PC) [n = 7]:* A hook was used to isolate the lateral portion of the sciatic nerve which was clamped in a hemostat for 30 s to create a 5 mm long crush injury.
3. *Peripheral Neuritis Trauma (PNT) [n = 24]:* Three 3-0 silk threads (Assut-UK), each 3 cm in length, were immersed overnight in complete Freund's adjuvant (CFA; 1 mg/ml) and loosely tied (1–2 mm apart) around the isolated lateral half of the sciatic nerve.
4. *Sham Control [n = 3]:* The sciatic nerve was exposed identically as for the other surgeries, but remained unmanipulated and fully intact.

#### 2.2.3 Wound closure and treatment post-surgery

Wounds were closed using a standard two-layer procedure whereby the subcutis layer was sutured with vicril 3-0 continuous stitches, followed by the skin layer being closed with continuous suture using 3-0 silk thread (Assut-UK). Following the wound closure, all pigs received marbofloxacin (10% w/v) (Marbocyl®, Vétoquinol UK Ltd., Buckingham, UK) administered via intramuscular (IM) injection into the neck muscle, at a total dose of 0.5 mL per pig. This treatment was continued for a period of 5 consecutive days. After recovering from anesthesia, the pigs were returned to their home pen.

### 2.3 Behavioral assessments

#### 2.3.1 Mechanical sensitivity

Mechanical sensitivity was assessed using various thickness nylon filaments (Touch Test (Von Frey) Sensory Evaluator Kit, model 58011, Stoelting Co., Wood Dale, IL, USA). The tests were performed in the home pens of each pig, using a modification of the up/down methods previously described for rodents (Chaplan et al., 1994). Various filaments with exertion to bend ranging from a minimum of 1 g (diameter = 0.229 mm; force = 9.80 mN) to a maximum of 60 g (diameter = 0.711 mm; force = 588.25 mN) were used. The filaments were applied on the dorsal area of the foot and on the external side of the knee three times each with a 5–10 s interval between applications. If withdrawal response was not achieved, the next thicker filament was applied, continuing until 60 g cutoff was achieved (most all uninjured pigs). If a withdrawal response was achieved, the next lower force filament was reapplied. Following six filament application tests alternating the filaments (up/down), the force required to achieve a withdrawal reaction was determined. Mechanical sensitivity testing was conducted on the day before surgery (−1) and on post-operative days (POD) ranging from 7 to 28 (see Table 2).

**Table 2:** Mechanical sensitivity response after sciatic insult (von Frey; g force).

	Day −1	POD 7	POD 10	POD 18	POD 28
SHAM	46.4 ± 8.3	60.0 ± 0.0	60.0 ± 0.0	53.2 ± 6.8	60.0 ± 0.0
FC	60.0 ± 0.0	60.0 ± 0.0	54.3 ± 13.9	1.7 ± 0.4^**#^	n/d
PC	57.6 ± 9.1	39.7 ± 28.2	50.3 ± 16.6	5.5 ± 3.4^**#^	6.5 ± 0.5^**#^
PNT	60.0 ± 0.0	3.7 ± 1.4^*#^	1.0 ± 0.0^**#^	5.1 ± 3.2^*#^	2.6 ± 2.3^**#^

^*^p < 0.05 vs. pre-surgery.

^**^p < 0.01 vs. pre-surgery.

^#^p < 0.05 vs. sham operated pigs.

#### 2.3.2 Tactile sensitivity

The tactile stimulus consisted of a 12.5 cm pigeon feather, which delivered light tactile stimulation upon gentle brush stroke across the dorsal area of the foot. Responder pigs expressed all three of the following behaviors: moving away, shaking and keeping the leg up, and guarding the leg for a period of 5 s. Tactile sensitivity testing was conducted on Day −1, and on POD ranging from 7 to 28, and the percentage of pigs that responded was recorded.

#### 2.3.3 Spontaneous pain behavior

The solitary performance and social behavior for each pig was scored during a 10-min observation period. Seven behavioral parameters were observed and recorded: 3 for solitary performance and 4 for social behavior, including standing posture/weight bearing, appearance (leg guarding, leg shaking), vocalization, and social behaviors (restlessness, agitation, aggression, isolation). Each behavioral parameter was graded (0–2) and the sum of all 7 parameters was considered the final score (Castel et al., 2016). Higher scores were indicative of more spontaneous pain behaviors in the pigs, which were recorded on the day before surgery, and on POD ranging from 3 to 28 (see Table 3).

**Table 3:** Spontaneous behavior after sciatic insult: pain and motor scores.

Pain Score	*Model*	*POD 3*	*POD 7*	*POD 10*	*POD 18*	*POD 28*
	Sham	0.6 ± 0.2	0.0 ± 0.0	0.0 ± 0.0	0.0 ± 0.0	0.0 ± 0.0
	FC	5.7 ± 1.2	7.7 ± 1.5	6.3 ± 1.6	6.0 ± 1.0	n/d
	PC	5.9 ± 1.2	7.3 ± 1.9	5.3 ± 1.5	5.3 ± 0.3	4.8 ± 1.0
	PNT	7.8 ± 2.1	6.4 ± 1.6	6.5 ± 1.0	7.0 ± 0.6	4 ± 0.4

Motor score	Sham	0.0 ± 0.0	0.0 ± 0.0	0.0 ± 0.0	0.0 ± 0.0	0.0 ± 0.0
	FC	4.0 ± 0.0	2.8 ± 0.6	3.0 ± 1.0	1.3 ± 1.6	n/d
	PC	4.0 ± 0.0	2.3 ± 1.0	1.4 ± 1.4	0.6 ± 0.4	0.8 ± 0.6
	PNT	1.0 ± 0.0	0.3 ± 0.4	0.1 ± 0.2	0.2 ± 0.4	0.2 ± 0.4

#### 2.3.4 Motor function

The ability of the pigs to use their leg properly was assessed by observing the pigs standing posture and their ability to walk properly (Castel et al., 2016). Motor function was graded from 0 to 2 points: 0 = normal; 1 = occasional flip of the foot; 2 = not able to keep the foot in the normal position. This grading system was used to assess foot position when standing and walking, thus the maximum possible score was 4 (severe motor dysfunction). Due to a loss of motor function and foot drag, all FC pigs were euthanized on POD 18 to prevent the development of secondary wounds. Therefore, no data was recorded for FC pigs on POD 28. Motor function assessments were performed on POD 3 to 28 on PC and PNT pigs (see Table 3).

### 2.4 Skin biopsy preparation

#### 2.4.1 Biopsy collection and sectioning

Immediately following euthanasia, an ∼0.5 × 1 cm^2^ skin biopsy was collected from painful skin locations on the dorsum of the foot ipsilateral to the sciatic nerve surgeries from all pigs (Table 1). A symmetrically located biopsy was also taken from the dorsal surface of the contralateral (unoperated) side of PNT1, PNT7, PNT14, and PNT21 pigs. The biopsies were immersion fixed in freshly-prepared 4% paraformaldehyde (in 0.1 M phosphate buffered saline; pH 7.4) for 4 h at 4 °C, rinsed in PBS at 4 °C, cryoprotected overnight in 30% sucrose/PBS, frozen, and cryostat sectioned at 14 µm thickness perpendicular to the epidermal surface as described previously (Pare et al., 2001, Pare et al., 2007, Albrecht et al., 2006, Albrecht et al., 2013, Bowsher et al., 2009). Sections were thaw mounted in serial order, alternating across at least 20 slides such that each slide contained sections from equally spaced intervals throughout the biopsy.

#### 2.4.2 Immunolabeling

Following previously published protocols, alternating slides of biopsy sections were processed for multi-molecular immunolabeling epifluorescence evaluation of cutaneous innervation and epidermal keratinocytes utilizing the ChemoMorphometric Analysis (CMA) platform developed by Integrated Tissue Dynamics, LLC (INTiDYN.com). All slides were processed for the specific immunolabeling as described below, and counterstained with 4′,6-diamidino-2-phenylindole (DAPI, Thermo Fisher, USA) to reveal cell nuclei (blue fluorescence).

#### 2.4.3 Innervation

One slide from each biopsy was processed to evaluate cutaneous innervation by double-labeling with antibodies against:

1. calcitonin gene-related peptide (CGRP; sheep polyclonal, 1:800, AbCam) which was revealed by subsequent donkey anti-sheep IgG conjugated to Cy3 (red fluorescence).
2. protein gene product 9.5 (PGP; rabbit polyclonal, 1:800 UltraClone) which was revealed by subsequent donkey anti-rabbit IgG conjugated to Alexa488 (green fluorescence). PGP is an enzyme (ubiquitin C-terminal hydrolase L1, UCHL1) which is concentrated in neuroendocrine secretory cells and the antibody robustly labels all peripheral neural innervation, including small caliber intraepidermal nerve fibers (IENF) that terminate among the vital keratinocytes.

**Fig. 1:**
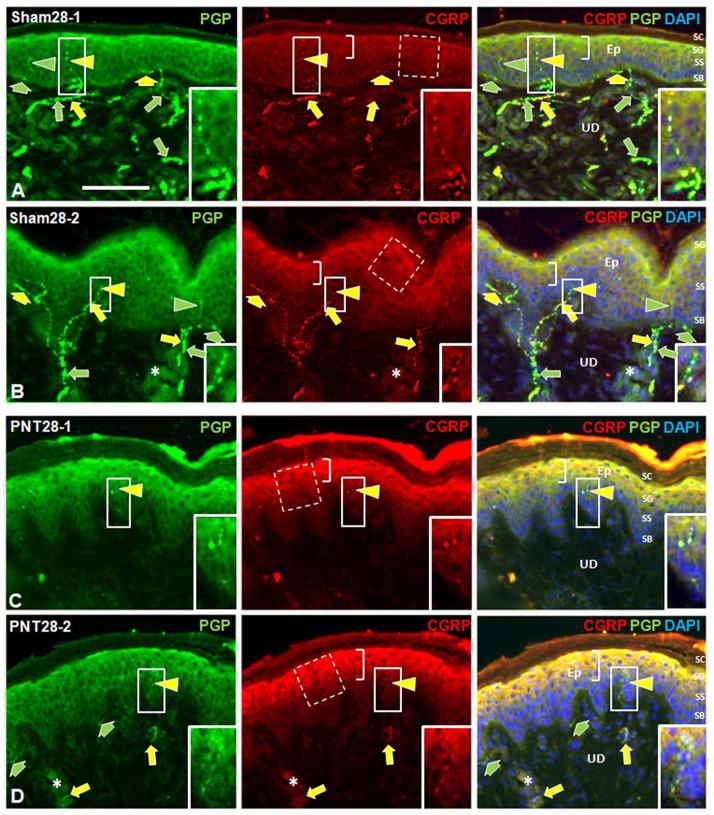
Representative images of double immunolabeling for PGP (green fluorescence, left column) and CGRP (red fluorescence, middle column) in ipsilateral biopsies from the dorsal hind foot of two Sham28 (A, B) and two PNT28 pigs (C, D). Merged images with DAPI nuclear staining (blue fluorescence) are shown in the right column. E, epidermis; UD, upper dermis; SB, Stratum Basalis; SS, Stratum Spinosum; SG, Stratum Granulosum; SC, Stratum Corneum. Scale bar = 100 μm. Arrowheads indicate immunolabeled neural profiles in the epidermis which are sensory intraepidermal nerve fiber (IENF) endings. Inserts in the solid line rectangles are 2× enlargements of endings shown in the smaller solid line rectangles, which are shown at a brighter and higher contrast. Broad arrows indicate immunolabeled neural profiles in the subepidermis, immediately subjacent to the epidermal basement membrane, which are a mix of individual fibers or small nerves containing two or more axon fibers. Long arrows indicate immunolabeled neural profiles in the upper dermis which are a mix of individual fibers and two or more fibers within small nerves, some of which are affiliated with small upper dermal blood vessels, particularly observed by concentrated DAPI labeling of cells in the vessel walls (asterisks). Nonpeptidergic neural profiles only labeled for PGP (green arrowheads and arrows), and peptidergic neural profiles double-labeled for CGRP and PGP (yellow arrowheads and arrows). Innervation at all levels was depleted following PNT28 injury compared with Sham28 biopsies. Middle panel brackets indicate the epidermal keratinocyte strata labeled for CGRP, which was more intense following PNT28 injury compared with Sham28 biopsies. Broken line rectangles represent the size and location of the sampling marquee that was systematically applied to quantify average CGRP immunofluorescence PI. (For interpretation of the references to colour in this figure legend, the reader is referred to the web version of this article.)

**Fig. 2:**
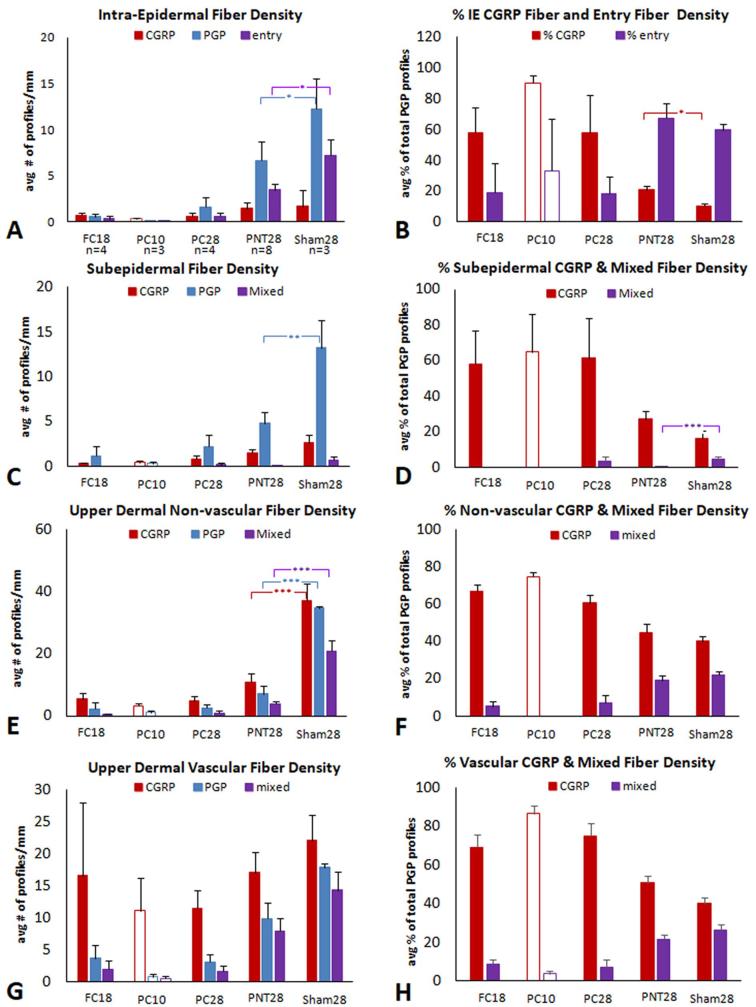
Average density and ratio measures of neural profiles (bars and SEM) characterized as peptidergic and nonpeptidergic for ipsilateral FC18, PC10, PC28, PNT28, and Sham28 biopsies (see Table 1, Part 1). PC10 bars are unfilled since they involve a purposefully shorter post-operative biopsy collection. ^*^p ≤ 0.05; ^**^p ≤ 0.01; ^***^p ≤ 0.005. (A, B) IENF density. (A) Red bars are the average peptidergic fiber density defined as profiles within the epidermis that co-label for CGRP and PGP. Blue bars are the average nonpeptidergic fiber density that only label for PGP. Purple bars are the average density of both types that have a discernible entry into the epidermis defined as a contact with or passage through the epidermal basement membrane. (B) Red bars are the average percentage of the total profiles that are peptidergic. Purple bars are average percentage of the total profiles that have an entry point. (C, E, G) Red bars are the average peptidergic fiber density defined as profiles that only co-label for CGRP and PGP. Blue bars are the average nonpeptidergic fiber density that only label for PGP. Purple bars are those profiles that can contain at least one peptidergic and one nonpeptidergic axon. (D, F, H) Red bars are the average percentage of the total profiles that are peptidergic. Purple bars are average percentage of the total profiles that have an entry point. (C, D) Subepidermal fiber density and ratio measures. (E, F) Upper dermal non-vascular fiber density and ratio measures. (G, H) Upper dermal vascular fiber density and ratio measures. (For interpretation of the references to colour in this figure legend, the reader is referred to the web version of this article.)

PGP immunolabeling reveals all types of cutaneous innervation, including the small-caliber unmyelinated C fibers, which are the vast majority, and lightly myelinated Aδ fibers of which both types are implicated in pain (Fig. 1). A contingent of these small-caliber fibers co-label with CGRP, a potent vasodilatory peptide, and are referred to as peptidergic fibers. Those small caliber fibers that label for PGP without CGRP are referred to as nonpeptidergic fibers. Quantification of the innervation is shown in Fig. 2, Fig. 5 (Supplementary Figs. S2 and S6).

#### 2.4.4 Epidermal keratinocytes

Based on from prior research, selected neurosignaling modulators were evaluated for alterations in immunolabeling among epidermal keratinocyte, including:

1. CGRP using the same slide of sections processed for the innervation.

Three additional slides from each biopsy were also prepared to detect keratinocyte immunolabeling with specific antibodies against:

2. voltage-gated sodium channel alpha subunit 1.7 (Nav1.7; rabbit polyclonal, 1:500, Alomone).
3. endothelin-1 receptor A (ETA; rabbit polyclonal, 1:500, Abcam).
4. endothelin-1 receptor B (ETB; rabbit polyclonal, 1:500, Abcam).

**Fig. 3:**
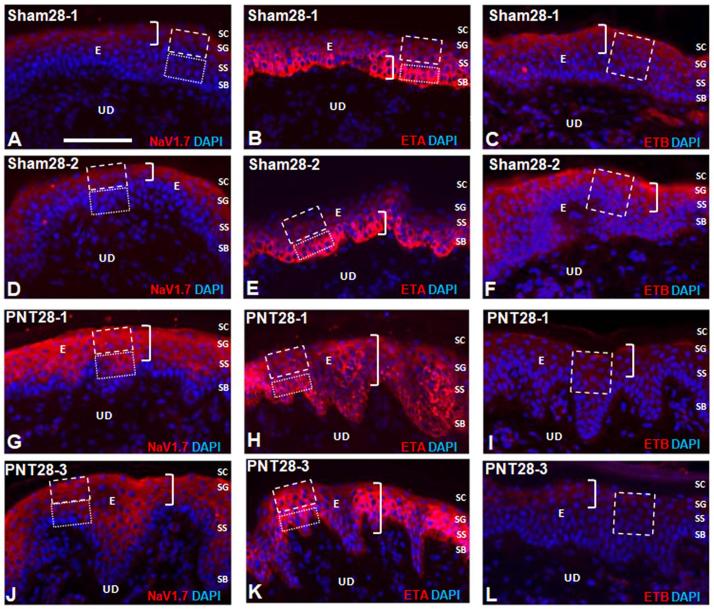
Representative images of Cy3 immunofluorescent labeling (red) for Nav1.7 (left column), ETA (middle column), and ETB (right column) in two Sham28 (A–F) and two PNT28 (G–L) ipsilateral biopsies. Cell nuclei are counterstained with DAPI (blue fluorescence). E, epidermis; UD, upper dermis; SB, Stratum Basalis; SS, Stratum Spinosum; SG, Stratum Granulosum; SC, Stratum Corneum. Scale bar = 100 μm. A,D,G,J. Nav1.7 immunolableing in Sham28 biopsies was concentrated in SG and upper SS (brackets in A, D) and increased in intensity as well as expanding through the full depth of SS, encroaching on SB in PNT28 biopsies (brackets in G, J). Quantification was based on repeated sampling of average PI in the upper and lower keratinocytes demarcated by the broken and dotted line marquees, respectively. (B, E, H, K) ETA expression in Sham28 biopsies was concentrated in SB (brackets in B, E) and expanded through the full depth of SG and SS in PNT28 biopsies (brackets in H, K). Quantification was based on repeated sampling of average pixel intensities in the SG and SS and in SB demarcated by the broken and dotted line marquees, respectively. (C, F, I, L) ETB expression in Sham28 biopsies was concentrated in SG and upper SS (brackets in C, F) and diminished in PNT28 biopsies (brackets in I, L). Quantification was based on repeated sampling of average pixel intensities in the SG and SS demarcated by the broken line marquees. (For interpretation of the references to colour in this figure legend, the reader is referred to the web version of this article.)

The Nav1.7, ETA, and ETB antibodies were revealed by subsequent donkey anti-rabbit IgG conjugated with Cy3 (Fig. 3). Keratinocyte expression of CGRP, Nav1.7, and ETA have each been implicated in mediating excitation (algesia), while keratinocyte expression of ETB has been implicated in mediating inhibition (analgesia) of sensory endings among the keratinocytes (Fig. 1).

**Supplementary Fig. S1:**
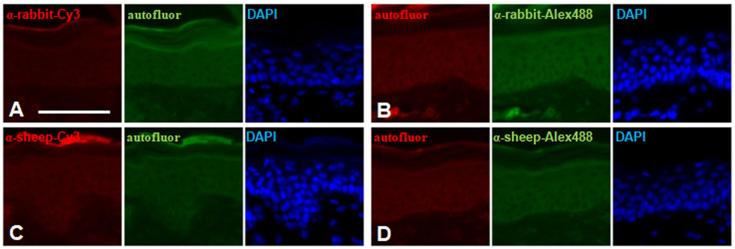
Separate channel images of the epidermal and upper dermal labeling only with donkey anti-rabbit (A, B) and donkey anti-sheep (C, D) secondary antibodies, omitting primary andtibodies and counter-labeling with DAPI (blue channel). (A, C) Secondary antibodies conjugated with Cy3 (red channel) combined with autofluorescence in the green channel. (B, D) Secondary antibodies conjugated with Alexa 488 (green channel) combined with autofluorescence in the red channel.

All primary and secondary antibodies utilized for this study have been validated for labeling specificity via previous studies of rats, monkeys, and humans. Particularly in mixed immunolabeling combinations, the similarity in labeling patterns across control/normal samples from various species, coupled with message expression and/or pharmacology has validated the immunolabeling results among keratinocytes (Khodorova et al., 2003, Zhao et al., 2008, Hou et al., 2011). The commercially available antibody preparations used were affinity purified with the antigen, and therefore as an additional control for secondary antibody non-specific reactivity, omission of primary antibodies was also performed on pig tissue (Fig. S1).

### 2.5 Skin biopsy analysis

The focus of our investigation was on thin-caliber cutaneous innervation implicated in pain (nociceptors) and on affiliated epidermal keratinocytes which have been shown to modulate the sensitivity of this innervation (Baumbauer et al., 2015, Moehring et al., 2018). For these evaluations, complete high-resolution epifluorescence digital montages of immunolabeled biopsy sections were captured using a computer linked Olympus BX51-WI microscope equipped with conventional fluorescence filters, a Hamamatsu ER DVC high-speed camera, and 3-axis motorized stage system interfaced with Neurolucida software (MBF Bioscience, Essex, VT). The data collection and CMA platform were performed under completely blinded conditions.

#### 2.5.1 Cutaneous innervation

##### 2.5.1.1 Innervation Organization

As documented previously, NeuroLucida routines were used to map the various types and features of CGRP and PGP labeled neural profiles detected in the epidermis and upper dermis across the entire width of three ∼ 1 cm wide sections equally spaced through each biopsy. As shown in Fig. 1, the papillary (upper) dermis consists of that part of the dermis located immediately deep to, and approximately the same thickness of the epidermis. It approximately corresponds to a layer composed of particularly compact collagen bundles, in contrast to the reticular (deep) dermis that is less compact and contains sweat glands and arteriole vasculature. In the epidermis and upper dermis, the labeled neural profiles consist of sensory endings, individual axons, and small nerves containing two or more axons. The small nerves are composed mostly of unmyelinated C fibers and some lightly myelinated Aδ fibers, that are mostly nonpeptidergic and some peptidergic. These nerves ascend through the reticular dermis and ramify in the papillary dermis where some individual fibers innervate interspersed capillaries and precapillary arterioles. Small branches of the nerves continue to ascend and ramify in a subepidermal plexus just beneath and parallel to the epidermal basement membrane, and most axons penetrate the basement membrane to enter and terminate as individual free sensory nerve endings in the epidermis (e.g., IENF).

##### 2.5.1.2 Innervation Quantification

To quantify the innervation, all labeled peptidergic and nonpeptidergic profiles in the epidermis, subepidermis, and upper dermis were mapped and counted across the full width of each section. Total counts were divided by the measured length of the evaluated epidermis to calculate the density as profiles/mm epidermal length. Labeled neural profiles are often referred to as fibers even though they are specifically sensory endings in the epidermis, or can be observed as individual axons or small nerves in subepidermal and upper dermal locations. Using this terminology, the following densities were quantified:

1. *Intra-Epidermal Nerve Fiber (IENF) Density* (Figs. 2A, 5A, S1A, S5A): These are profiles of sensory endings in the epidermis, categorized as complete fiber endings or fragments (caused by plane of sectioning), and as peptidergic or nonpeptidergic. IENF which were seen to be in contact with the basement membrane were further categorized epidermal entry points.
2. *Subepidermal Fiber Density* (Figs. 2C, 5C, S1B, S6A): These are peptidergic and nonpeptidergic profiles of subepidermal small nerves and individual fibers. All contiguous nerve profiles were mapped and further categorized as mixed nerves if they contained individual peptidergic and nonpeptidergic axons.
3. *Upper Dermal Fiber Density* (Figs. 2E, 5E, S1C, S7A): These are peptidergic and nonpeptidergic profiles of upper dermal small nerves and individual fibers. All contiguous nerve profiles were mapped and were further categorized as mixed nerves if they contained individual peptidergic and nonpeptidergic axons. Two analyses were performed on the upper dermal nerve counts. For the Part 1 analyses (Figs. 2 and S1), the upper dermal fiber profiles were subdivided into those that were associated with upper dermal capillaries and pre-capillary arterioles, identified by clusters of DAPI labeled nuclei (Figs. 2G, H, S1D). For the Part 2 analyses (Figs. 5E and S7), all upper dermal fiber profiles were included. Thus, for analyses of PNT28 pigs, the upper dermal fiber densities were split into nonvascular and vascular affiliated profiles for Part 1, whereas these were combined for Part 2.
4. *Upper Dermal Vascular Fiber Density* (Only for Part 1 analyses; Figs. 2G and S5G): These are peptidergic, nonpeptidergic, and mixed upper dermal profiles of small nerves and individual fibers that were only associated with upper dermal arterioles and capillaries as identified by clusters of DAPI labeled nuclei.

#### 2.5.2 Epidermal keratinocyte neurochemistry

##### 2.5.2.1 Epidermal organization

The epidermis is composed of 3 layers of vital (live) keratinocytes structurally defined as Stratum Basalis (SB), Stratum Spinosum (SS), and Stratum Granulosum (SG) (Fig. 1, Fig. 3). Keratinocytes are continually replaced from progenitors in SB and are displaced and differentiate through SS and SG, whereupon the keratinocytes produce high lipid content, degrade the nuclei, and cornify to form the dead Stratum Corneum (SC) at the most external surface of the skin.

##### 2.5.2.2 Immunolabel quantification

The immunolabeling pixel intensity (PI) for NaV1.7, ETA, CGRP, and ETB was quantified from microscopic camera-captured epifluorescent images with the camera capture settings held constant for each immunolabel across all samples. PI was collected using an equal sized sampling marquis from 10 equally-spaced sample locations across the entire length of the epidermis, collected from 3 individual sections equally spaced through each biopsy (Photoshop CS3, Adobe Systems, San Jose). The particular epidermal location strategies utilized for collecting PI for each of the immunolabels was based on the distribution in biopsies from healthy human specimen (devoid of chronic pain afflictions), and therefore regarded as normal. Similar distributions were confirmed by qualitative observations in the sham and contralateral pig biopsies.

**Fig. 4:**
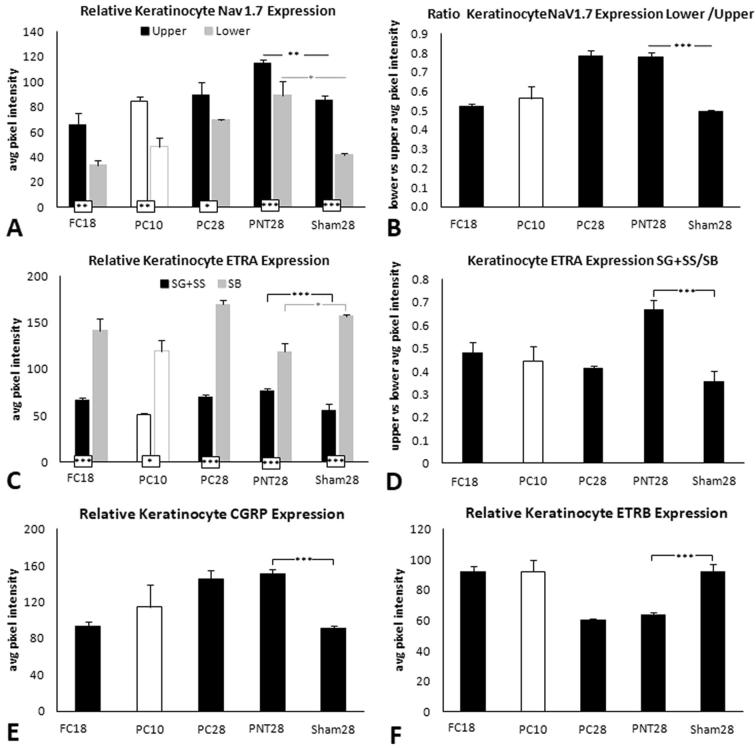
Keratinocyte average immunofluorescent pixel intensity (PI) from FC18, PC10, PC28, PNT28, and Sham28 for Nav1.7 (A), ETA (C), CGRP (E), and ETB (F). ^*^p ≤ 0.05; ^**^p ≤ 0.01; ^***^p ≤ 0.005. (B) Ratio of lower to upper average PI for Nav1.7 (black and gray bars respectively). (D) Ratio of combined SG and SS to SB average PI for ETA (black and gray bars respectively).

**Supplementary Fig. S2:**
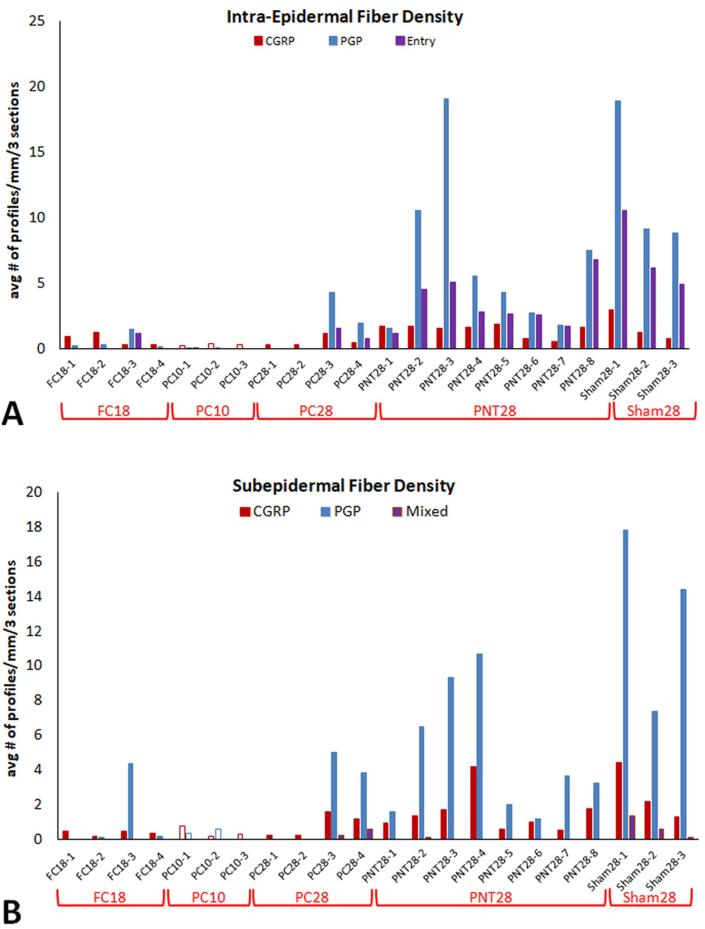

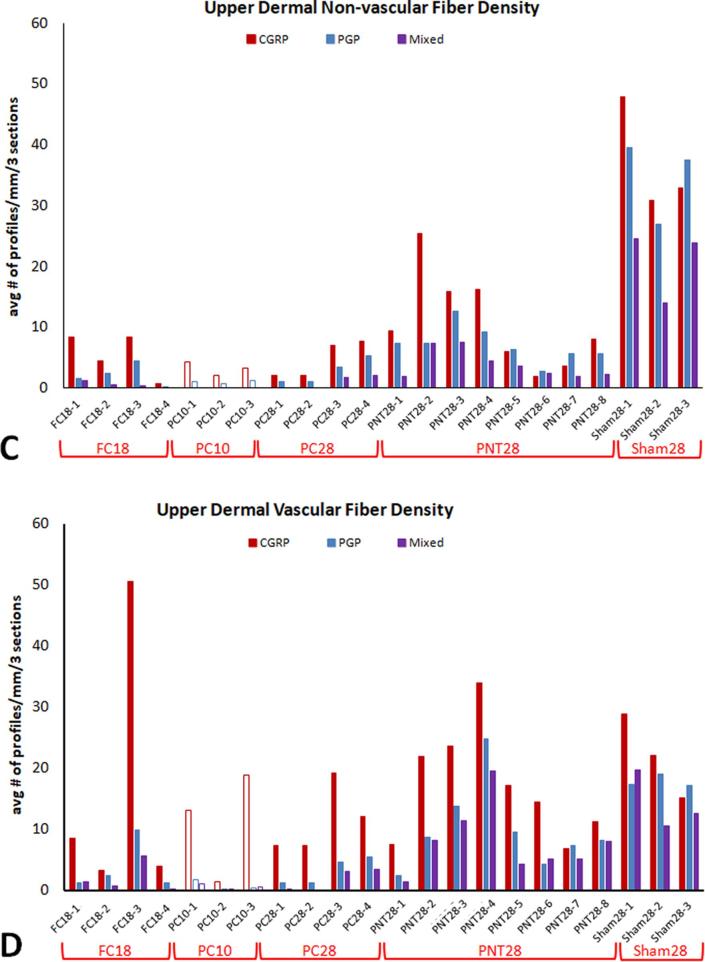
Average density of neural profiles (bars and SEM) characterized as peptidergic and nonpeptidergic for each ipsilateral FC18, PC10, PC28, PNT28, and Sham28 biopsy (see Table 1, Part 1). PC10 bars are unfilled since they involve a purposefully shorter post-operative time. (A) IENF density. Red bars are the average peptidergic fiber density defined as profiles within the epidermis that co-label for CGRP and PGP. Blue bars are the average nonpeptidergic fiber density as profiles that only label for PGP. Purple bars are the average density of both types that have a discernible entry into the epidermis defined as a contact with or passage through the epidermal basement membrane. (B–D) Subepidermal (B), upper dermal non-vascular (C), and upper dermal vascular (D). Red bars are the average peptidergic fiber density defined as profiles that only co-label for CGRP and PGP. Blue bars are the average nonpeptidergic fiber density as profiles that only label for PGP. Purple bars are those profiles that can contain at least one peptidergic and one nonpeptidergic axon.

**Supplementary Fig. S3:**
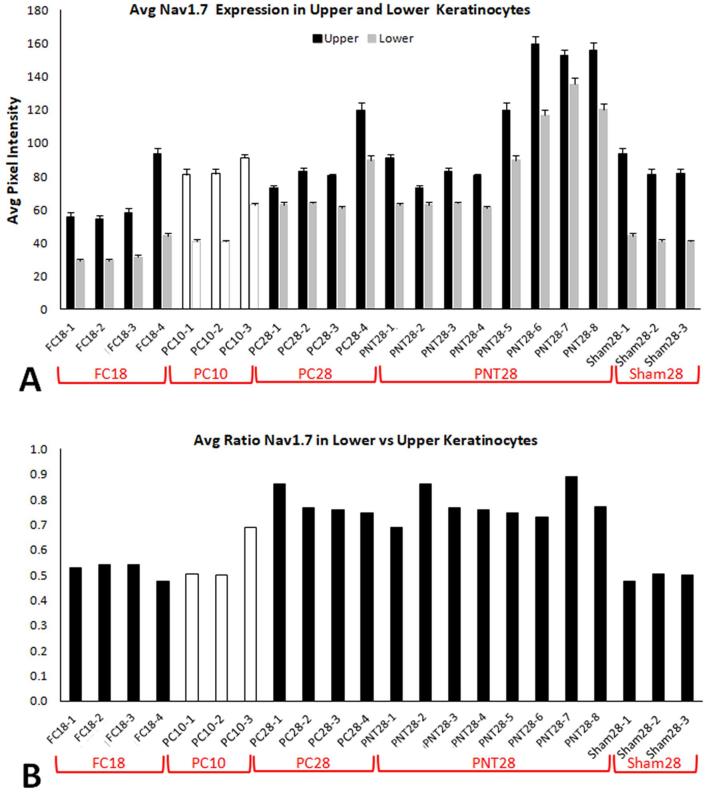
(A) Average keratinocyte Nav1.7 fluorescent pixel intensities (PI) in ipsilateral biopsies among upper and lower keratinocytes (black and gray bars respectively) for each FC18, PC10, PC28, PNT28, and Sham28 pig. (B) Ratio of lower divided by upper average Nav1.7 keratinocyte PI for each pig.

**Supplementary Fig. S4:**
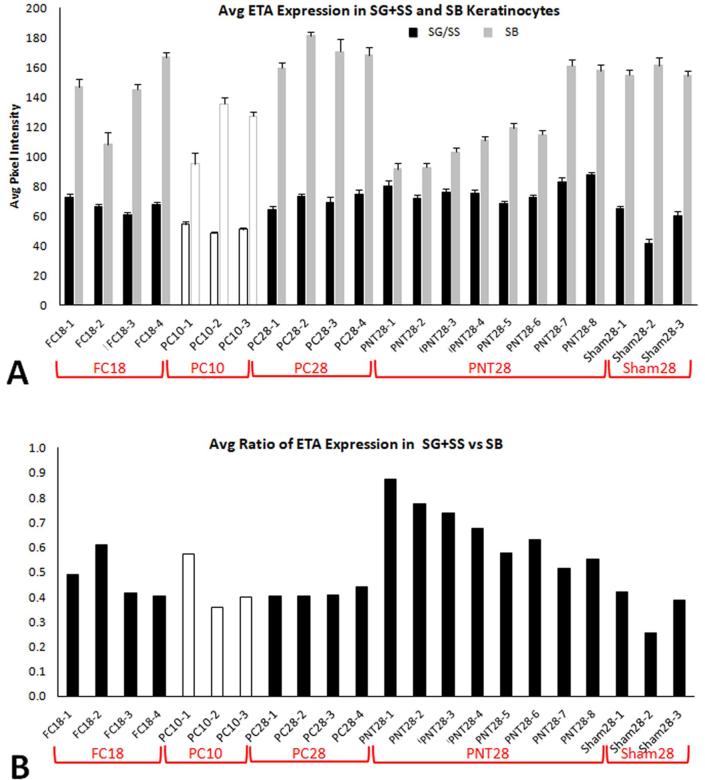
(A) Average keratinocyte ETA fluorescent PI in ipsilateral biopsies among SG and SS, and SB keratinocytes (black and gray bars respectively) for each FC18, PC10, PC28, PNT28, and Sham28 pig. (B) Ratio of SG and SS divided by SB average Nav1.7 keratinocyte PI for each pig.

1. *NaV1.7:* NaV1.7 immunolabeling is normally stratified to SG and the upper SS, and occasionally observed among SB. Therefore, for measures of the epidermis, a dual NaV1.7 sampling strategy was used to quantify keratinocyte PI using one sampling marquee for repeated sampling in the pixels captured in the approximate upper half and another marquee for the lower half (Figs. 3A, D, G, J; 4A; 6; S2; S8).
2. *ETA:* ETA immunolabeling is normally stratified almost exclusively to SB. Therefore, a dual ETRA sampling strategy was used to quantify keratinocyte PI by using one sampling marquee for repeated sampling in SB and another marquee for SS and SG combined (Figs. 3B, E, H, K; 4C; 7; S3, S9).
3. *CGRP:* CGRP immunolabeling is normally diffusely distributed across SG and among SS, gradually diminishing towards SB, where it is rarely observed. Therefore, for measures of the epidermis, a single CGRP sampling strategy was used to quantify keratinocyte PI using pixels captured in the marquee spanning SG and SS combined, excluding the SB. (Figs. 1, 4E, 8, S4A, S10).
4. *ETRB:* ETRB immunolabeling is normally diffusely distributed across SG and SS, gradually diminishing in intensity towards SB, where it is rarely observed. Therefore, a single ETRB sampling strategy was used to quantify keratinocyte PI using pixels captured in the marquee spanning SG and SS combined, excluding SB (Figs. 3C, F, I, L; 4F; 9; S4B, S11).

#### 2.5.3 Statistical analysis

The PNT model was developed in pigs as a large animal model for experimentally inducing pain through a proximal nerve irritation, without the overt physical injury of a transection or crush, that would be more relevant to human peripheral neuritis due to proximal nerve irritation, inflammation, and/or mild constriction. The first objective of the statistical comparisons was to assess the long term impact of the PNT insults. Therefore, statistical analyses using unpaired T-tests were conducted on data from PNT28 pigs in comparison to Sham28 pigs. The second objective, was to assess the time-course when the various PNT pathologies, observed on POD 28, could be detected after injury, and also to determine the status of the contralateral biopsies taken from the same pig. Statistical comparisons between ipsilateral and contralateral biopsies from the same pigs on POD 1, 7, 14, and 21 were performed using paired *T*-tests. Ipsilateral results on POD 7, 14, 21, and 28 were individually compared to results on POD 1 using unpaired *T*-tests.

## 3 Results

### 3.1 Behavioral results

#### 3.1.1 Mechanical sensitivity

Pigs that underwent FC and PC exhibited significantly increased sensitivity to VF stimuli on the foot compared with sham or pre-surgery pigs between POD 10 and 18, as expressed by the low force eliciting withdrawal (Table 2). On POD 28, the significant increase in sensitivity to VF stimuli on the foot remained for the PC pigs. PNT pigs exhibited significantly increased sensitivity to VF filaments which was present by POD 7, the earliest test day after surgery. Significant mechanical sensitivity remained throughout the POD 28 study period for the PNT pigs (Table 2).

#### 3.1.2 Tactile sensitivity

On POD 7, 100% of the pigs from all three peripheral nerve trauma models responded to light tactile stimulation. On POD 28, 80% of the pigs in the PNT group responded to the light tactile stimuli (i.e., 4 out of 5 tested pigs), indicative of long-standing tactile hypersensitivity.

#### 3.1.3 Spontaneous pain behavior

Spontaneous pain behaviors were not observed in any pigs at Day −1 (0.0 ± 0.0; data not shown in Table 3), and changes in spontaneous pig behavior were observed as early as POD 3 in all three peripheral nerve trauma models (Table 3). There were increased pain scores following FC, PC, or PNT, indicative of ongoing pain when standing and/or walking. The most common spontaneous response following nerve crush injury (FC or PC) was flipping of the foot (90% of nerve crush injured pigs exhibited foot flip within 24 h to POD 7). In contrast, only one PNT-injured pig exhibited slight foot flip on or after POD 7.

#### 3.1.4 Motor function

Following FC or PC, pigs exhibited significant motor dysfunction, as evidenced by maximum motor scores on POD 3. Overall, the motor function scores decreased over the study period as most pigs improved their ability to stand or to walk (Table 2). Following PNT-injury, there was almost no change from baseline in ability to walk or to stand using the injured leg. Notably, PNT pigs rarely exhibited only minor foot flip and none had secondary injuries.

### 3.2 ChemoMorphometric profile of PNT impact (study part 1)

#### 3.2.1 Qualitative innervation observations

Examples of ipsilateral biopsy immunolabeling of innervation for PGP and CGRP on POD 28 are shown from two Sham28 and two PNT28 pigs in Fig. 1. Consistent with prior published observations in other species including human, the Sham28 biopsies (Fig. 1A, B) had individual intraepidermal nerve fibers (IENF) that were nonpeptidergic labeling only for PGP (green arrowheads) and peptidergic that double-labeled for CGRP and PGP (arrowheads). The IENF were supplied from small subepidermal (broad arrows) and upper dermal (narrow arrows) nerves containing two or more axons, that could be all nonpeptidergic (green arrow) or a mix of nonpeptidergic and peptidergic (yellow arrows). Some individual axons in the upper dermis may be terminating among the dispersed fibroblasts. Some small nerves and individual axons in the upper dermis are in close proximity to, and presumably innervate, capillaries and precapillary arterioles (asterisks). In the PNT28 biopsies (Fig. 1C, D), the innervation of the papillary dermis, subepidermis, and epidermis was obviously depleted compared to that of the Sham28 biopsies.

#### 3.2.2 Quantitative innervation analyses

Quantitative group averages of the innervation for study part 1 are shown in Fig. 2. Individual pig results are shown in supplementary data (Fig. S2). While significant differences were observed among the group averages, some PNT28 pigs had innervation similar to that of Sham28 pigs, while others were severely impacted.

##### 3.2.2.1 IENF density

In Sham28 biopsies (Fig. 2A, B), about 10% of all IENF were peptidergic as evidenced by co-labeling for CGRP and PGP (red bars) with the remaining being nonpeptidergic as evidenced by labeling for PGP only (blue bars). About 60% of all IENF had detectable entry points (purple bars). Compared to Sham28 controls (Fig. 2A), a significant reduction occurred in PNT28 biopsies among the average density of the nonpeptidergic nerve fibers (blue bars, p ≤ 0.05) as well as in the density of all fibers that had a detectable entry point (purple bars, p ≤ 0.05). No significant change occurred among the peptidergic fibers (red bars). Consequently, the percentage of peptidergic fibers was significantly higher (∼20%) in the PNT28 biopsies compared to Sham28 (Fig. 2B, red bars, p ≤ 0.05). The percentage of total fibers with entry points was not significantly different. Within these averages and significant differences, some PNT28 were comparable to Sham28s with others more severely impacted. Compared with PNT28, the FC18, PC10, and PC28 biopsies had a far greater loss of total IENF, similarly among the nonpeptidergic, with the majority (∼60%) of the few remaining fibers being peptidergic (Fig. 2A, B).

##### 3.2.2.2 Subepidermal fiber density

Very few subepidermal profiles contained a mix of peptidergic and nonpeptidergic fibers in Sham28 biopsies (Fig. 2C, purple bars). About 15% of all subepidermal profiles in Sham28 biopsies were entirely peptidergic nerves or individual fibers (Fig. 2C, D; red bars) with nearly all the rest nonpeptidergic (blue bars). Consistent with IENF density, a highly significant decrease occurred among the subepidermal innervation in PNT28 biopsies, particularly among the nonpeptidergic profiles (blue bars, p ≤ 0.01). Similarly, the relative preservation of peptidergic profiles (red bars) resulted in a virtual absence of mixed nerves (purple bars, p ≤ 0.005). Compared with PNT28, the FC and PC lesions again resulted in an even more severe innervation loss, similarly among the nonpeptidergic compared to the peptidergic profiles (Fig. 2C, D).

##### 3.2.2.3 Non-vascular upper dermal fiber density

The upper dermal non-vascular fiber densities had the most consistency across the individual pigs for each type of nerve insult (Fig. S2C). The Sham28 biopsies had a fairly even density and proportion of peptidergic and nonpeptidergic profiles (Fig. 2E, red and blue bars respectively) with a high proportion having a mix of peptidergic and nonpeptidergic fibers (Fig. 2E, F; purple bars). In PNT28 biopsies, an equally highly significant (p ≤ 0.005) and equally proportioned decrease occurred across the peptidergic, nonpeptidergic, and mixed profile densities (Fig. 2E, F). Compared with PNT28, a more severe depletion occurred in the FC and PC biopsies, which impacted the density of nonpeptidergic more than the peptidergic profiles.

##### 3.2.2.4 Vascular upper dermal fiber density

The vasculature affiliated innervation in the upper dermis, especially the peptidergic contingent, remained mostly preserved in the PNT28 biopsies and did not test as significantly less than that in Sham28 biopsies (Fig. 2G, H). The peptidergic fibers were also remarkably preserved in FC and PC biopsies whereas the nonpeptidergic component was severely depleted.

#### 3.2.3 Quantitative keratinocyte immunolabeling

Previous research has indicated that Nav1.7, ETA, and CGRP expression in epidermal keratinocytes represent potential excitatory (algesic) modulators of innervation terminating in or near the epidermis, and keratinocyte immunolabeling for Nav1.7, ETA, and CGRP are increased in several human chronic pain afflictions (Zhao et al., 2008, Hou et al., 2011). Additionally, previous research has indicated that ETB expression in epidermal keratinocytes represents a potential inhibitory (analgesic) modulator of innervation terminating in or near the epidermis, and keratinocyte immunolabeling for ETB is decreased in several painful human conditions (Albrecht and Rice, unpublished observations).

##### 3.2.3.1 Keratinocyte Nav1.7

In Sham28 biopsies, Nav1.7 epidermal keratinocyte immunolabeling was observed primarily in SG and outer SS (brackets; Fig. 3A, 3D). In PNT28 biopsies, Nav1.7 epidermal immunolabeling increased in intensity and spread to the inner SS keratinocytes, with only faint labeling in SB (brackets; Fig. 3G, J). The average PI of Nav1.7 immunolabeling was quantified both for SG and outer SS combined (broken line marquee), referred to as upper, and for inner SS and SB combined (dotted line marquee), referred to as lower. Group average PI for the different types of sciatic nerve manipulations are shown in Fig. 4A and B with individual averages for each pig in Fig. S3. Paired *T*-tests revealed that Nav1.7 immunolabeling was significantly higher in the upper compared to lower keratinocytes for all FC, PC, PNT, and Sham groups. Consistent with the qualitative indications, in PNT28 biopsies, Nav1.7 immunolabeling was significantly increased in the upper keratinocytes (P < 0.01) and lower keratinocytes (P < 0.05) in comparison to those in Sham28 biopsies (Fig. 4A), with a highly significant proportionately greater increase occurring among the lower keratinocytes (Fig. 4B, p < 0.005). Nav1.7 immunolabeling was also significantly increased in lower, but not upper, keratinocytes of PC28 biopsies, and was not increased in upper or lower keratinocytes from FC18 and PC10 biopsies compared to that of Sham28.

##### 3.2.3.2 Keratinocyte ETA

In Sham28 biopsies, ETA keratinocyte immunolabeling was observed to be intense throughout SB (brackets in Fig. 3B, E), with only a faint labeling among more superficial keratinocytes. In PNT28 biopsies, ETA keratinocyte immunolabeling substantially increased throughout SG and SS, while decreasing in SB (Fig. 3H, K). The average PI of ETA immunolabeling was quantified both for SG and SS combined (broken line marquee), referred to as upper, and for SB alone (dotted line marquee), referred to as lower. Group average PI for the different types of sciatic nerve manipulations are shown in Fig. 4C and D with individual averages for each pig in Fig. S4. Paired *T*-tests revealed that ETA immunolabeling was significantly increased in the lower compared to upper keratinocytes for all FC, PC, PNT, and Sham groups. Consistent with the qualitative observations, in PNT28 biopsies, ETA PI was significantly increased in the upper keratinocytes (p < 0.005) and significantly decreased in lower keratinocytes (p < 0.05) compared with Sham28 biopsies (Fig. 4C), with a highly significant proportionately greater increase among the upper keratinocytes (Fig. 4D, p < 0.005). ETA expression in upper keratinocytes also appears to have increased in FC18 and PC28 biopsies, but without a decrease in lower keratinocytes.

##### 3.2.3.3 Keratinocyte CGRP

**Supplementary Fig. S5:**
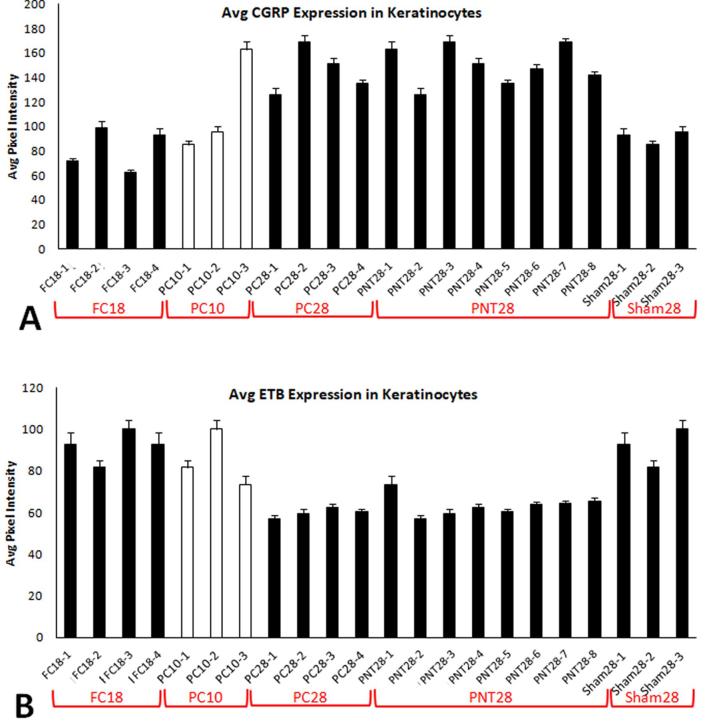
(A) Average keratinocyte CGRP fluorescent PI in ipsilateral biopsies from each FC18, PC10, PC28, PNT28, and Sham28 pig. (B) Average keratinocyte ETB fluorescent PI in ipsilateral biopsies from each pig.

In Sham28 biopsies, CGRP immunolabeling was observed to be primarily among epidermal SG and SS (upper) keratinocytes, and increased in intensity in this location in the PNT28 biopies (Fig. 1, middle column brackets). An increase in CGRP PI, quantified over this location (Fig. 1, broken line marquee), was highly significant in PNT28 biopsies compared to Sham28 (Fig. 4D, p < 0.005), and was comparably increased among PC28 biopsies but not those of FC18 or PC10. The CGRP quantification was highly consistent across the individual pigs (Fig. S5A).

##### 3.2.3.4 Keratinocyte ETB

In Sham28 biopsies, ETB immunolabeling was observed to be primarily among epidermal SG and SS (upper) keratinocytes, and decreased in intensity in this location in the PNT28 biopsies (Fig. 4C, F, I, L; middle column brackets). A decrease in ETB PI, quantified over this location (Fig. 4C, F, I, L; broken line marquees), was highly significant in PNT28 biopsies compared to Sham28 (Fig. 4D, p < 0.005), and was comparably decreased among PC28 biopsies but not those of FC18 or PC10. The ETB quantification was highly consistent across the individual pigs (Fig. S5B).

### 3.3 Development of PNT ChemoMorphometric impacts (study part 2)

**Fig. 5:**
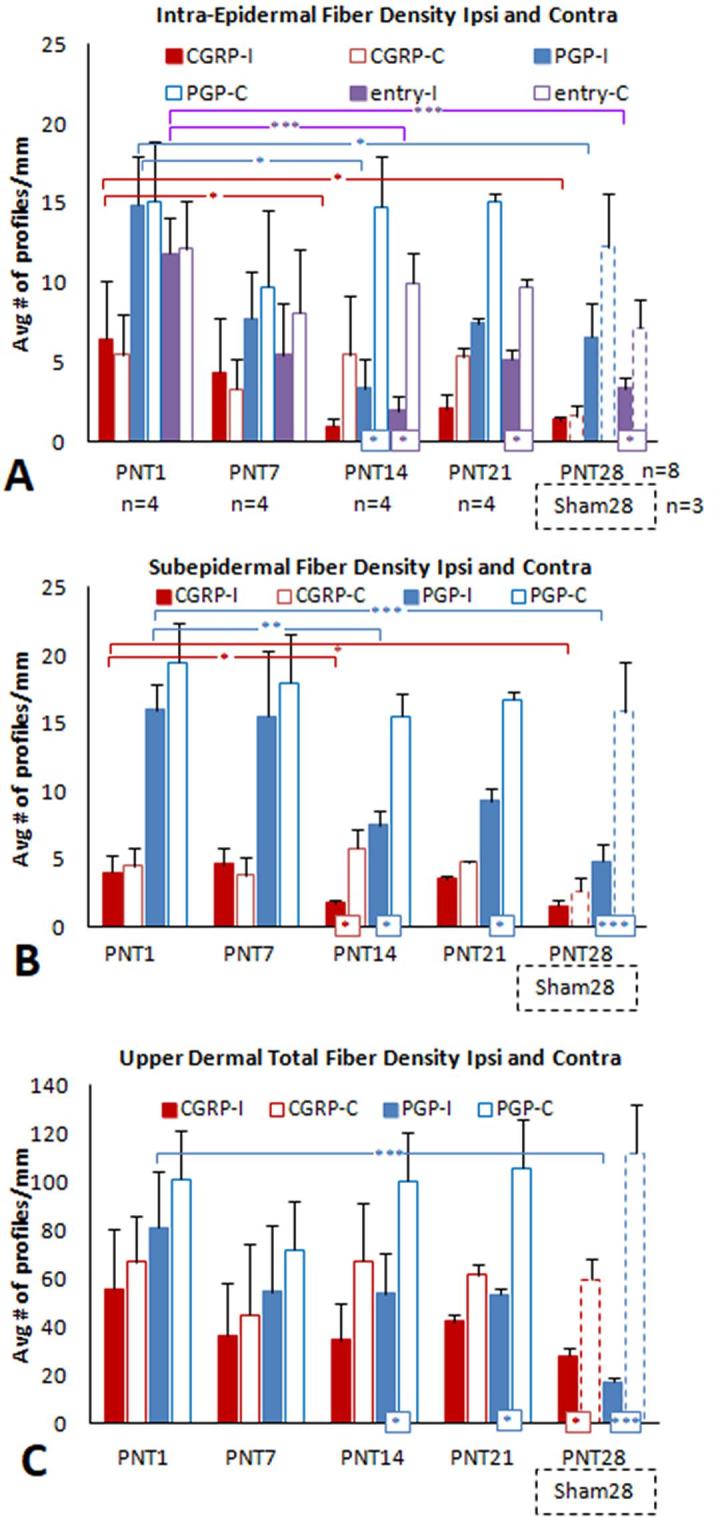
Average density of neural profiles characterized as peptidergic and nonpeptidergic from ipsilateral and contralateral PNT1, PNT7, PNT14 and PNT21 biopsies (see Table 1, Part 2). Results from ipsilateral PNT28 and Sham28 (broken line bars) biopsies are included from Fig. 2A. ^*^p ≤ 0.05; ^**^p ≤ 0.01; ^***^p ≤ 0.005. (A) IENF density. Red bars are the average peptidergic fiber density defined as profiles within the epidermis that co-label for CGRP and PGP. Blue bars are the average nonpeptidergic fiber density as profiles that only label for PGP. Purple bars are the average density of both types that have a discernible entry into the epidermis defined as a contact with or passage through the epidermal basement membrane. (B, C) Subepidermal fiber density (B) and upper dermal total vascular and non-vascular fiber density (C). Red bars are the average peptidergic fiber density defined as profiles that only co-label for CGRP and PGP. Blue bars are the average nonpeptidergic fiber density as profiles that only label for PGP. Purple bars are those profiles that can contain at least one peptidergic and one nonpeptidergic axon. (For interpretation of the references to colour in this figure legend, the reader is referred to the web version of this article.)

**Fig. 6:**
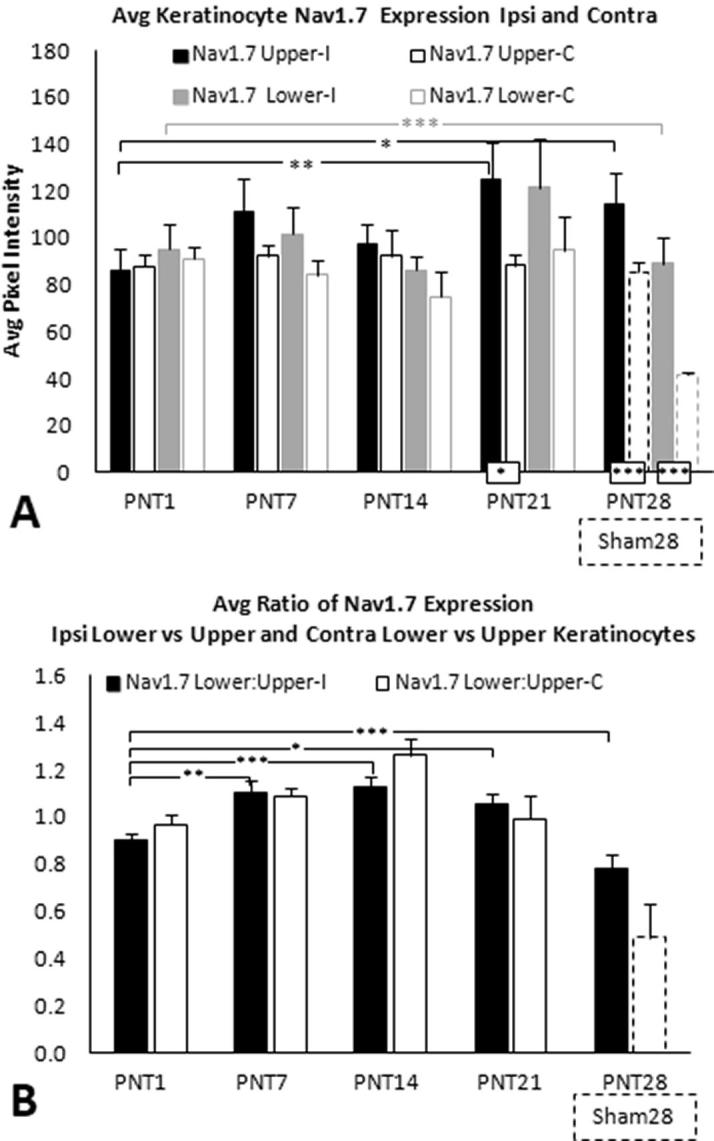
(A) Average keratinocyte Nav1.7 PI among upper and lower keratinocytes for ipsilateral biopsies (solid black and gray bars, respectively) and contralateral biopsies (open black and gray bars, respectively) from PNT1, PNT7, PNT14, and PNT 21 pigs (see Table 1, Part 2). (B) Ratios of lower divided by upper average keratinocyte Nav1.7 PI for ipsilateral and contralateral biopsies (solid and open bars, respectively). (A) and (B) show results from average ipsilateral Nav1.7 PI among upper and lower epidermal keratinocytes in PNT28 biopsies (solid black and gray bars, respectively) and ipsilateral Sham28 biopsies (open broken line black and gray bars, respectively), included from Fig. 4A and B.

**Fig. 7:**
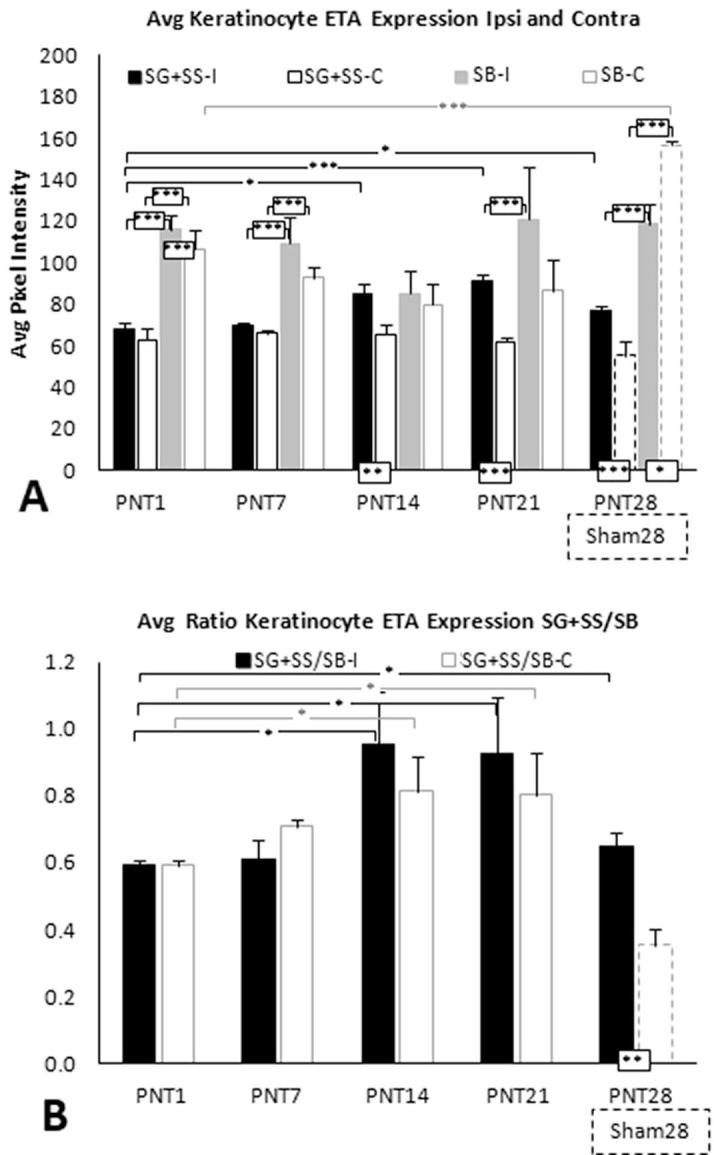
(A) Average keratinocyte ETA PI among SG and SS and among SB keratinocytes for ipsilateral biopsies (solid black and gray bars, respectively) and contralateral biopsies (open black and gray bars, respectively) from PNT1, PNT7, PNT14, and PNT 21 pigs (see Table 1, Part 2). (B) Ratios of SG and SS divided by SB average keratinocyte ETA PI for ipsilateral and contralateral biopsies (solid and open bars, respectively). (A) and (B) show results from average ipsilateral ETA PI in SG and SS and in SB epidermal keratinocytes in PNT28 biopsies (solid black and gray bars, respectively) and ipsilateral Sham28 biopsies (open broken line black and gray bars, respectively), included from Fig. 4C and D.

**Fig. 8:**
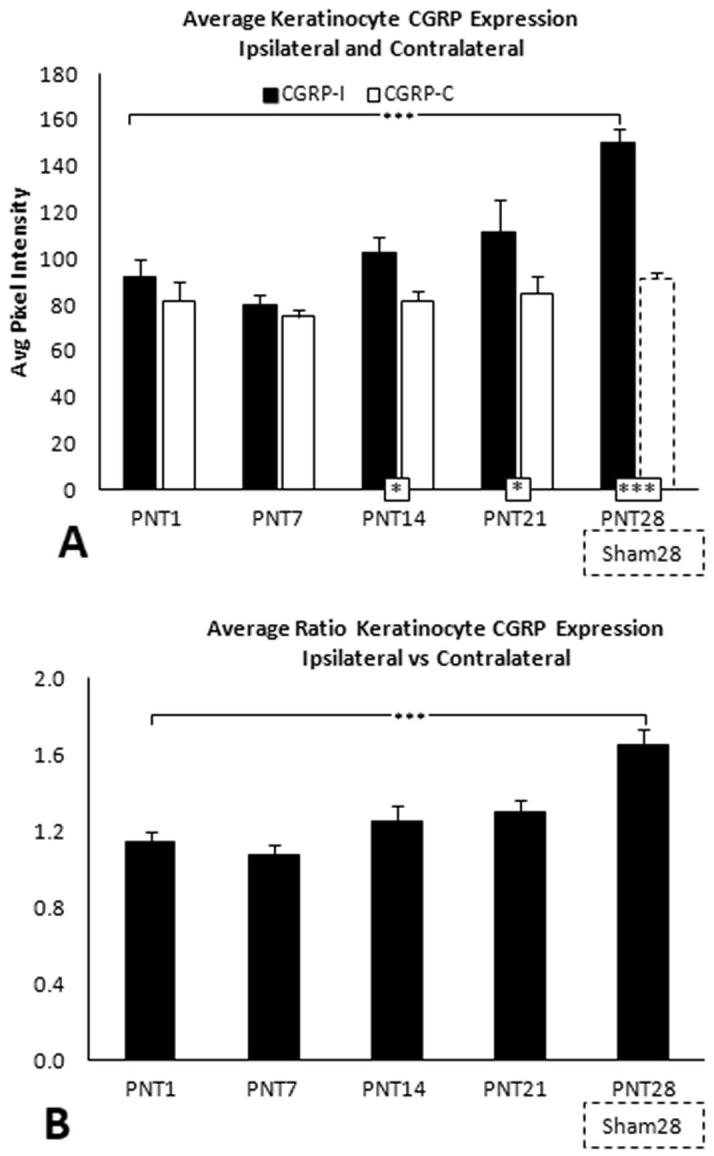
(A) Average keratinocyte CGRP PI for ipsilateral and contralateral biopsies (solid and open bars, respectively) from PNT1, PNT7, PNT14, and PNT 21 pigs (see Table 1, Part 2). (B) Ratios of ipsilateral divided by contralateral average CGRP PI. (A) and (B) show results for ipsilateral PNT28 biopsies and Sham28 biopsies (broken lines), included from Fig. 4E.

**Fig. 9:**
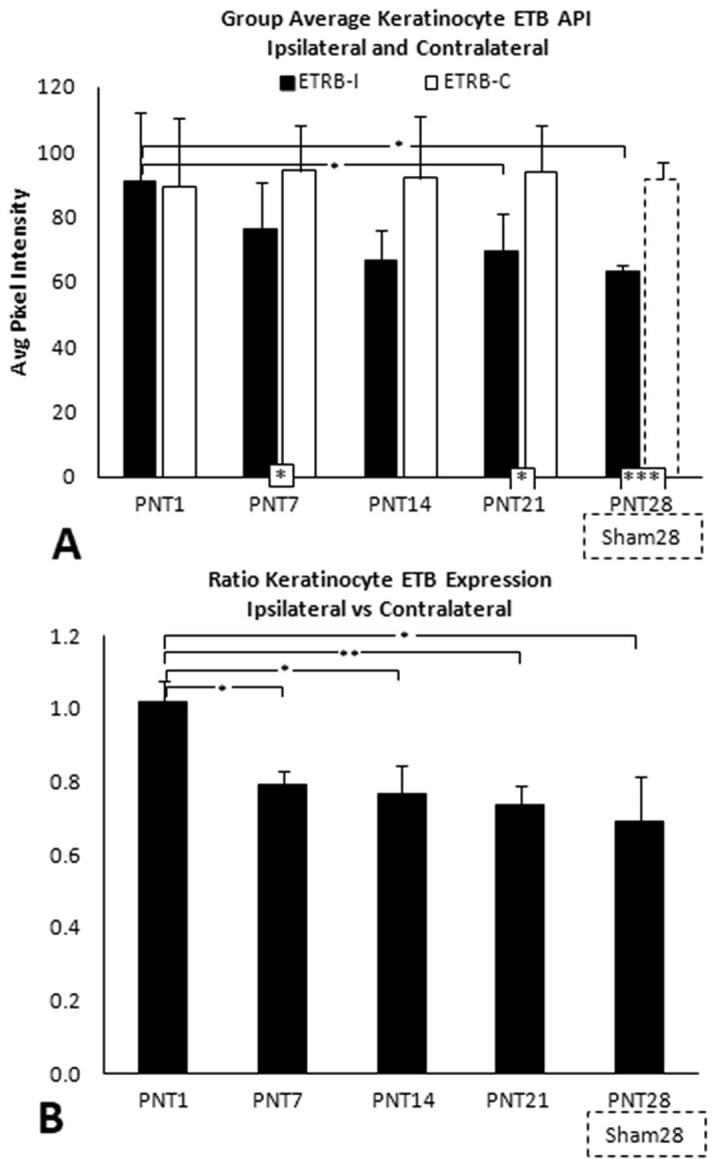
(A) Average keratinocyte ETB PI for ipsilateral and contralateral biopsies (solid and open bars, respectively) from PNT1, PNT7, PNT14, and PNT 21 pigs (see Table 1, Part 2). (B) Ratios of ipsilateral divided by contralateral average ETB PI. (A) and (B) show results for ipsilateral PNT28 biopsies and Sham28 biopsies (broken lines), included from Fig. 4F.

**Supplementary Fig. S6:**
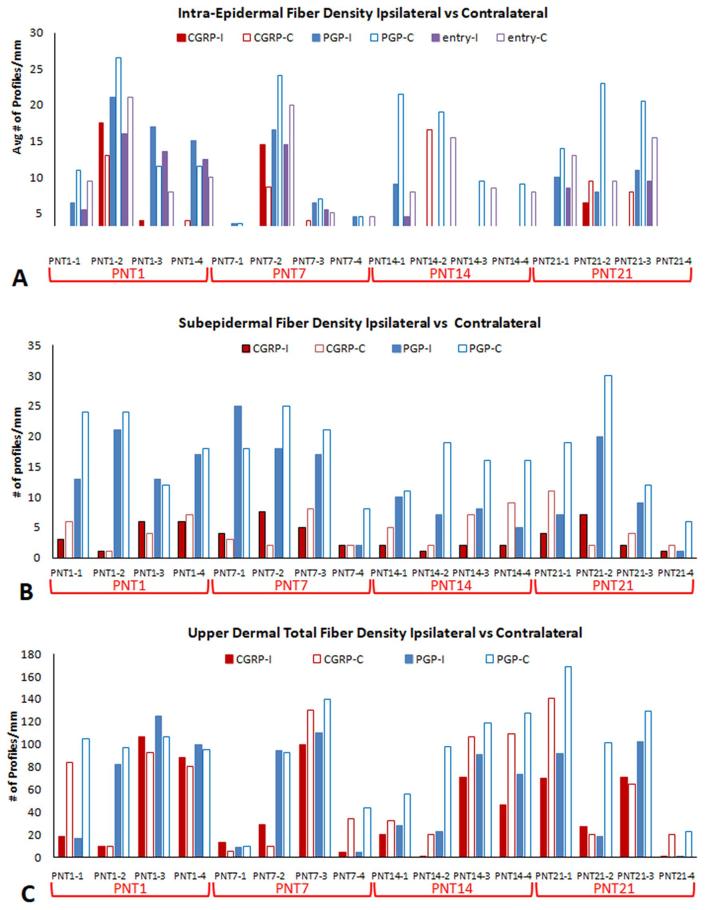
Average density of neural profiles (bars and SEM) characterized as peptidergic and nonpeptidergic for each ipsilateral (solid bars) and contralateral biopsy (open bars) from each PNT1, PNT7, PNT14, and PNT21 pig (see Table 1, Part 2). (A) IENF density. Red bars are the average peptidergic fiber density defined as profiles within the epidermis that co-label for CGRP and PGP. Blue bars are the average nonpeptidergic fiber density as profiles that only label for PGP. Purple bars are the average density of both types that have a discernible entry into the epidermis defined as a contact with or passage through the epidermal basement membrane. (B, C) Subepidermal (B) and total upper dermal. Red bars are the average peptidergic fiber density defined as profiles that only co-label for CGRP and PGP. Blue bars are the average nonpeptidergic fiber density as profiles that only label for PGP. Purple bars are those profiles that can contain at least one peptidergic and one nonpeptidergic axon.

**Supplementary Fig. S7:**
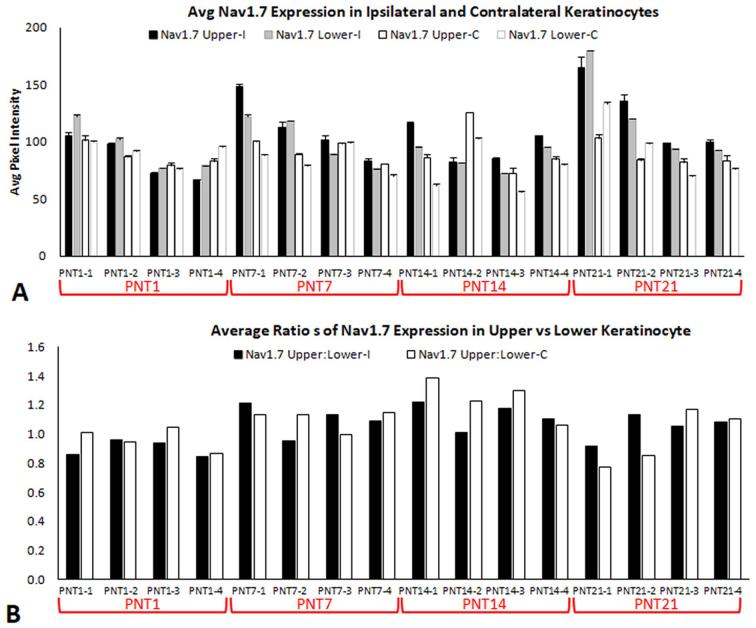
(A) Average Nav1.7 fluorescent PI among upper and lower keratinocytes in ipsilateral (solid black and gray bars, respectively) and contralateral biopsies (open black and gray bars, respectively) from each PNT1, PNT7, PNT14, and PNT 21 pig (see Table 1, Part 2). (B) Ratios of lower divided by upper average keratinocyte Nav1.7 PI in ipsilateral and contralateral biopsies (solid and open bars, respectively).

**Supplementary Fig. S8:**
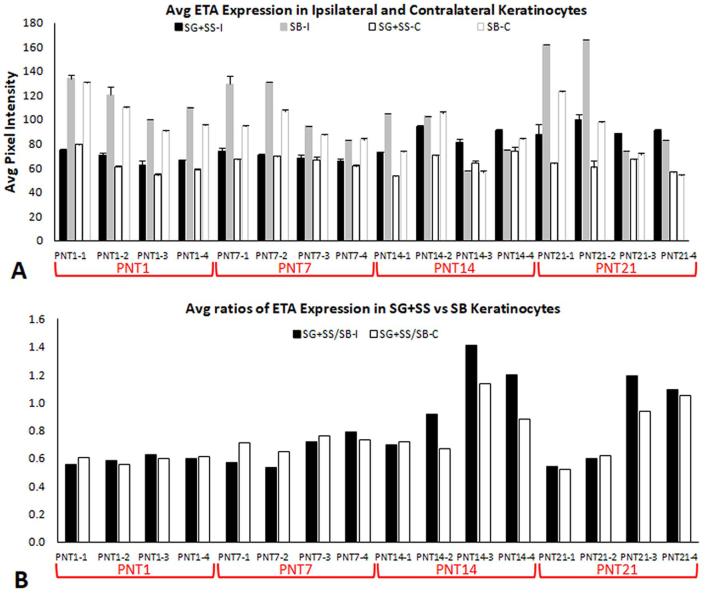
(A) Average ETA fluorescent PI among SG and SS and among SB keratinocytes in ipsilateral (solid black and gray bars, respectively) and contralateral biopsies (open black and gray bars, respectively) from each PNT1, PNT7, PNT14, and PNT 21 pig. (see Table 1, Part 2). (B) Ratios of SG and SS divided by SB average keratinocyte ETA PI in ipsilateral and contralateral biopsies (solid and open bars, respectively).

**Supplementary Fig. S9:**
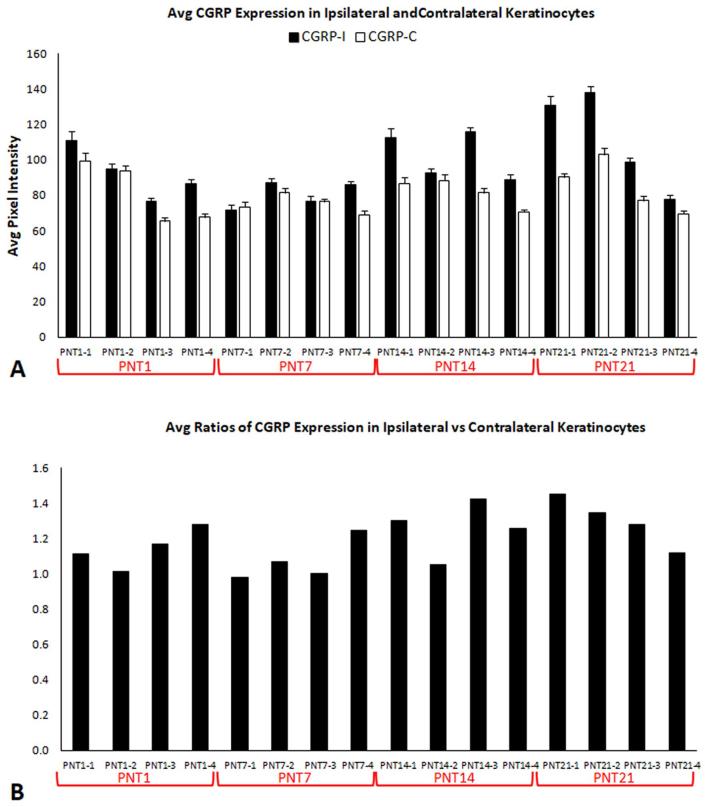
(A) Average keratinocyte CGRP fluorescent PI in ipsilateral and contralateral biopsies (solid and open bars, respectively) from each PNT1, PNT7, PNT14, and PNT21 pig (see Table 1, Part 2). (B) Ratios of ipsilateral divided by contralateral average CGRP PI.

**Supplementary Fig. S10:**
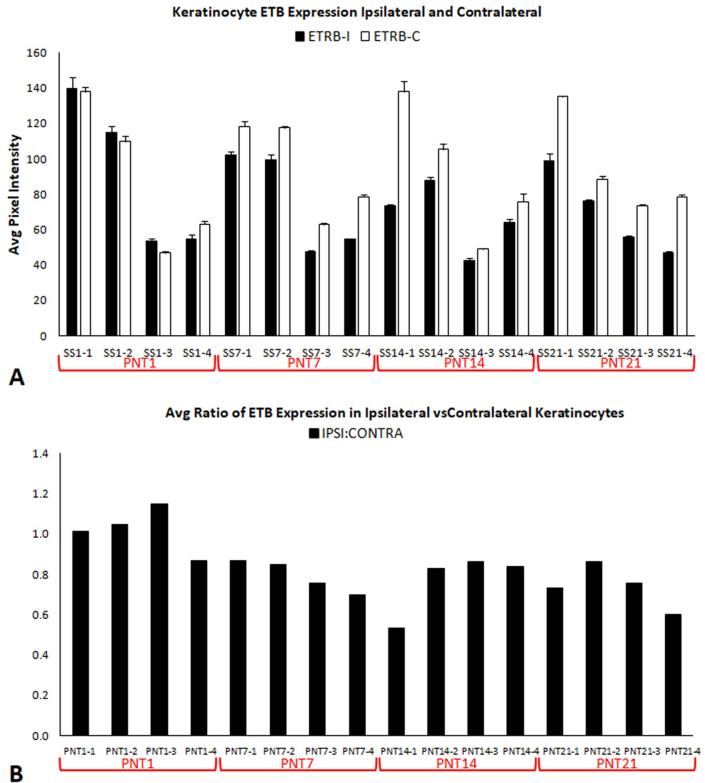
(A) Average keratinocyte ETB fluorescent PI in ipsilateral and contralateral biopsies (solid and open bars, respectively) from each PNT1, PNT7, PNT14, and PNT21 pig (see Table 1, Part 2). (B) Ratios of ipsilateral divided by contralateral average ETB PI.

Having established an impact of sciatic nerve PNT insult on the cutaneous innervation and keratinocyte immunolabeling by POD 28 in study part 1, the objective of study part 2 was to assess the time course of PNT induced pathologies on POD 1, 7, 14, and 21. These assessments also included an analysis of a mirror image biopsy from the contralateral unoperated side of each pig. Average results for each POD are shown in Fig. 5, Fig. 6, Fig. 7, Fig. 8, Fig. 9 which include the study part 1 PNT28 results for ipsilateral comparisons, while the Sham28 are used as contralateral comparisons. Individual averages for each pig are shown in Figs. S6–S10.

#### 3.3.1 Quantitative innervation analyses

##### 3.3.1.1 IENF density

IENF densities of peptidergic fibers (CGRP/PGP), nonpeptidergic fibers (only PGP), and epidermal fiber entry points for ipsilateral (I) and contralateral (C) biopsies for each group of PNT are shown in Fig. 5A, with individual biopsy averages shown in Fig. S6A. Among animal group sizes n = 4, considerable variability occurred among the individual pig biopsies for ipsilateral peptidergic and nonpeptidergic fibers, especially at PNT1 and PNT7. Some animals showed evidence of severe depletion compared to others, indicating that the PNT insult may be impacting the epidermal innervation within one day and especially during the first week post-op (Fig. S6A). As compared to the average of PNT1 biopsies, significant reductions of both the average peptidergic and nonpeptidergic fibers (p < 0.05) and especially among epidermal entry points (p < 0.005) were detected among the PNT14 biopsies, consistent with that observed among PNT28 biopsies (Fig. 5A). The reduction of entry points on the ipsilateral compared to the contralateral biopsies is significant (p < 0.05) among the PNT14 and PNT21 biopsies, as observed between PNT28 and Sham28 biopsies. Interestingly, the densities and proportions of peptidergic to nonpeptidergic fibers, as well as entry points, are especially high in both the ipsilateral and contralateral PNT1 biopsies as compared with those from other ipsilateral and contralateral PNT biopsies, being significantly higher for ipsilateral PNT1 compared to PNT28 biopsies (p < 0.05), with an indication that contralateral PNT1 are also higher compared to Sham28 biopsies. This data suggests that the IENF, especially those that are peptidergic, may undergo a transient increase in PNT1 biopsies.

##### 3.3.1.2 Subepidermal fiber density

Average subepidermal densities of peptidergic fibers and nonpeptidergic fibers for ipsilateral (I) and contralateral (C) biopsies for groups of PNT are shown in Fig. 5B, with individual biopsy averages in Fig. S6B. In contrast to IENF results, the subepidermal fiber densities were much more consistent among individual biopsies from the PNT groups (Fig. S6B). As compared to PNT1 biopsies, significant reductions of both the average peptidergic (p < 0.05) and nonpeptidergic fiber densities (p < 0.001) were detected for the PNT14 biopsies consistent with that observed for PNT28 (Fig. 5B).

##### 3.3.1.3 Upper dermal fiber density

Average upper dermal densities of peptidergic fibers and nonpeptidergic fibers for ipsilateral (I) and contralateral (C) biopsies for groups of PNT are shown in Fig. 5C, with individual biopsy averages in Fig. S6C. The average ipsilateral peptidergic and nonpeptidergic upper dermal fiber density appeared to be highest for PNT1 slightly dropping among PNT7, PNT14, and PNT21 (Fig. 5C). However, only in PNT28 biopsies was a significant decrease of the nonpeptidergic fibers (p < 0.005) observed. Contralateral upper dermal fiber densities were virtually identical across PNT1, PNT7, PNT14, and PNT21 biopsies, and were comparable to Sham28 biopsies.

#### 3.3.2 Quantitative keratinocyte neurochemical analyses

##### 3.3.2.1 Keratinocyte Nav1.7

In PNT1 biopsies, the average keratinocyte Nav1.7 immunolabeling PI in the lower and upper portions of the ipsilateral epidermis were both comparable to levels in the contralateral (control) side. Some indication of an increase on the ipsilateral side was already beginning on PNT7 and was significantly increased among the upper keratinocytes in ipsilateral PNT21 biopsies (p < 0.01), which was sustained among PNT28 (p < 0.05) (Fig. 6A; see Fig. S7 for individual pig data). Among PNT7, PNT14, and PNT21 biopsies, the average Nav1.7 PI among upper and lower epidermal keratinocytes remained comparable to that of PNT1 biopsies, and ipsilateral Sham28 controls. Surprisingly, Nav1.7 PI had a highly significant decrease in Sham28 biopsies compared to that in any of the contralateral biopsies from PNT1-PNT21 (p < 0.005, Fig. 6A). The average ratios of lower to upper epidermal Nav1.7 PI revealed significant increases at PNT7 (p < 0.01), PNT14 (p < 0.001), and PNT 21 (p < 0.05) compared with PNT1 (Fig. 6B).

##### 3.3.2.2 Keratinocyte ETA

In PNT1 biopsies, the average keratinocyte ETA immunolabeling PI in SG and SS (upper) and in SB (lower) of the ipsilateral epidermis were both comparable to their levels in the contralateral (control) side, with a highly significant greater intensity in SB (p < 0.005) (Fig. 7A; see Fig. S8 for individual pig data). ETA PI was significantly increased in SG and SS in ipsilateral PNT14 (p < 0.05), PNT21 (p < 0.005), and PNT28 (p < 0.05) biopsies compared to that in PNT1 and PNT7 biopsies and in comparison to the corresponding contralateral biopsies and ipsilateral Sham28 (Fig. 7A). Average ETA PI in SB remained comparable from PNT1 to PNT28 (Fig. 7A). Based on proportions in PNT1 biopsies, a significant increase occurred (p < 0.05) in the relative intensity of ETA PI in SG and SS compared to SB beginning in PNT14 biopsies, which is sustained through PNT28. Surprisingly, this significant increase occurred among contralateral PNT14 and PNT 21, but was not observed in ipsilateral Sham28 biopsies (Fig. 7B).

##### 3.3.2.3 Keratinocyte CGRP

In PNT1 biopsies, the average keratinocyte CGRP immunolabeling PI was comparable to that on the contralateral biopsies. Ipsilateral CGRP PI was increased compared with contralateral by PNT14 through PNT28 (vs sham) and was significantly increased in PNT28 biopsies compared with PNT1 (p < 0.005) (Fig. 8A; see Fig. S9 for individual pig data). Average contralateral CGRP PI remained nearly identical through PNT21, and was comparable to that in the ipsilateral Sham 28. Consequently, a significant increase in ratio of ipsilateral to contralateral CGRP PI was observed at PNT28 (Fig. 8B).

##### 3.3.2.4 Keratinocyte ETB

In PNT1 biopsies, the average keratinocyte ETB immunolabeling PI was comparable between ipsilateral and contralateral (Fig. 9A; see Fig. S10 for individual pig data). Average contralateral ETB PI levels remained nearly identical through PNT21, and were comparable to that in ipsilateral Sham28. Average ETB PI levels in the ipsilateral PNT7 and PNT21 biopsies were significantly lower compared to the contralateral controls (p < 0.05) (and were strongly trended at PNT14), and in ipsilateral PNT28 biopsies compared to Sham28 (p < 0.005). Ipsilateral ETB PI among PNT14 and PNT28 was significantly lower than at PNT1 (p < 0.05). Consequently, the ratios of ipsilateral to contralateral (and PNT28 to Sham28), were all significantly lower than at PNT1 (Fig. 9B).

## 4 Discussion

The development of successful therapeutics to treat nearly all forms of human NP with known involvement of cutaneous innervation has been hampered by the lack of translatable animal models, especially large animals having metabolic and physical features comparable to humans (Rice et al., 2008, Henze and Urban, 2010, Swindle et al., 2012). MD Biosciences, Inc. has developed a modified unilateral sciatic nerve PNT model in pigs that produces sustained NP behaviors consistent with those observed in human pain patients (Castel et al., 2016). This current study was performed to determine if the cutaneous innervation and epidermal keratinocytes of the dorsal foot of these PNT insult model pigs showed cutaneous pathologies consistent with those observed in a variety of human NP afflictions, including a case of severe CRPS in the hand and forearm triggered by proximal rotator cuff surgery (Holland et al., 1997, Polydefkis et al., 2001, Albrecht et al., 2006, Obermann et al., 2008, Vlckova-Moravcova et al., 2008, Weis et al., 2011, Hoeijmakers et al., 2012, Grone et al., 2014, Divisova et al., 2016, Albrecht et al., 2018). Particularly important is that the skin of pigs has a glabrous structure and innervation comparable to that of humans, including multiple layers of epidermal keratinocytes, even in pig hairy skin where, like humans, follicles are widely spaced (Rukwied et al., 2008, Hirth et al., 2013).

Here, we have further characterized the pig PNT model and have identified two important cutaneous pathologies in that are also observed in humans with chronic NP: 1) a reduction of small caliber C and Aδ innervation, particularly to the epidermis, and 2) alterations in the stratified excitatory and inhibitory neural signaling properties in epidermal keratinocytes. The reduction of small caliber epidermal innervation (i.e., SFN), presumed to be the source of nociceptor input, presents a paradox that is partially reconciled by electrophysiological evidence indicating that remaining innervation becomes hyperactive (Ochoa et al., 2005, Kleggetveit et al., 2012, Serra et al., 2014). However, in humans, NP can also occur without a reduction in small-fiber epidermal innervation, and small-fiber reductions can occur without NP symptoms, such as nonpainful diabetic neuropathy and pain-free recovery from acute herpes zoster (Petersen et al., 2002, Petersen et al., 2010, Reda et al., 2013, Albrecht et al., 2018). To date, little is known about the proportions of sensory fiber subtypes impacted in various types of peripheral neuropathies or changes in their inherent signaling properties, nor how alterations in the target compartment cells (i.e., epidermis, vasculature) may account for painful versus nonpainful outcomes in humans.

Comprehensive characterization of the pathologic changes associated with the pig PNT model provides considerable additional insight into the nature and etiology of neuritis-like pathologies, which may underlie small-fiber neuropathic insult. The current work included PNT comparisons to FC and PC insults which damage motor innervation and can interfere with assessments of pain behaviors. Clearly, the PNT insult model resulted in less innervation loss as compared to either FC or PC injuries. However, across all the models, both peptidergic and nonpeptidergic innervation was reduced at all cutaneous levels, especially the epidermis and subepidermis, validating the PNT insult as a human-like NP model. Importantly, peptidergic profiles were disproportionately spared, particularly those affiliated with upper dermal capillaries and precapillary arterioles. Current unpublished research at INTiDYN indicates preferential survival of peptidergic microvascular fibers in biopsies from our previous human NP studies (FL Rice and PJ Albrecht, unpublished observations). Thus, the preferential survival of the peptidergic fibers found in the current pig PNT insult model may also be a contributor to human chronic NP mechanisms. Additionally, the upper dermal innervation and particularly that associated with the microvasculature was particularly less impacted in the PNT28 biopsies compared with the FC or PC injuries, being comparable in some pigs to Sham28 biopsies, further validating the pig model as representative of human NP condition pathologies.

Examining the development of decreased sensory innervation after PNT insult revealed that significant and consistent loss of IENF occurs between POD7 and POD14, with a more gradual loss in the subepidermis and upper dermis through POD 21. Thus, a potential two-week therapeutic window exists after injury whereby interventions to partially prevent loss of innervation could be implemented. Importantly, PNT insult did not have a significant impact on the innervation in contralateral biopsies, which were similar to that in ipsilateral Sham28 biopsies at all time points. Overall, epidermal innervation density was more varied compared with subepidermal and upper dermal innervation among comparable biopsies from different pigs with the same insults, including the ipsilateral Sham28 biopsies (see also (Petersen et al., 2010)). Therefore, subepidermal and upper dermal innervation densities may provide more reliable measures of pathologies related to peripheral neuropathies than epidermal innervation densities alone.

Evidence from our work and others has now demonstrated that epidermal keratinocytes respond to and integrate cutaneous stimuli through autocrine/paracrine interactions which have both algesic (excitatory) and analgesic (inhibitory) neurosignaling properties that modulate or directly activate innervation within or adjacent to the epidermis (Khodorova et al., 2003, Ibrahim et al., 2005, Cannon et al., 2007, Zhao et al., 2008, Dussor et al., 2009, Kwan et al., 2009, Mandadi et al., 2009, Radtke et al., 2010, Hou et al., 2011, Baumbauer et al., 2015, Pang et al., 2015, Moehring et al., 2018). For example, we have previously shown that in normal rat glabrous skin, ETB-mediated keratinocyte release of β-endorphin likely has an inhibitory modulation on peptidergic innervation that expresses the µ-opioid receptor (Khodorova et al., 2003). Furthermore, under normal healthy conditions, these neurochemical signaling properties remain differentially stratified and are recapitulated during the process of keratinization, as cells mature from SB through SS and SG to become the corneocytes at the extreme epidermal surface. Importantly, among a variety of human NP pathologies conditions, we have now demonstrated altered immunolabeling among keratinocyte neurosignaling mediators, including an increase in epidermal algesic properties and a decrease in analgesic properties. That these altered keratinocyte patterns remain long after the inciting injury indicates that the pathologic keratinocyte patterns are also recapitulated and likely modulate the activity of remaining cutaneous innervation. Similar long-standing changes in epidermal keratinocyte properties have also been induced in the glabrous hindpaw skin of rats by proximal nerve insults (Hou et al., 2011).

The current results support an important mechanistic role of epidermal keratinocyte alterations in chronic NP, and demonstrates that the pig sciatic nerve PNT insult model develops epidermal keratinocyte pathologies among the neural signaling properties that closely resemble those identified in human NP conditions (Zhao et al., 2008, Dussor et al., 2009, Hou et al., 2011, Barr et al., 2013, Albrecht et al., 2018). For example, PNT resulted in increased expression of Nav1.7, ETA, and CGRP, which are all implicated in nociceptor excitatory algesic mechanisms, as well as a decrease in ETB, which is implicated in inhibitory analgesic mechanisms (Khodorova et al., 2003). These keratinocyte alterations likely contribute to remaining nociceptor hyperactivity both through increased algesic and reduced analgesic modulation (Rice and Albrecht, 2008, Albrecht and Rice, 2010). Surprisingly, these changes only occurred in the PC and PNT models, but did not occur after FC injury. This suggests that not only can pathologies be induced among epidermal keratinocytes after a proximal nerve insult, but also that the induction requires more sparring of innervation than occurs after FC injury. Thus, the recapitulating pathological neural signaling patterns among the epidermal keratinocytes, as well as normal keratinocyte patterning, may involve a dynamic interaction with the innervation subtypes.

Interestingly, the development of the keratinocyte pathologies appears to occur over a different time course for each marker. For example, increased keratinocyte Nav1.7 was already apparent by POD 1 in what may be a rapid response to PNT insult. Moreover, as compared to the impact of ipsilateral Sham28 surgeries, it appears that Nav1.7 may also be increased among biopsies contralateral to the PNT insult. By contrast, PNT-induced changes in keratinocyte immunolabeling for ETA, ETB, and CGRP only occurred ipsilaterally and with a delayed time course. A significant decrease in analgesic keratinocyte ETB expression was first detectable on POD 7 and remained decreased through POD 28. A significant increased in algesic keratinocyte CGRP and ETA expression was first evident at POD 14 and persisted through POD 28. As well, both algesic mediators CGRP and ETA are also mitogens that may be impacting the dynamics of keratinocyte production and wound healing. In particular, the changed expression of ETA in superficial keratinocytes seems especially interesting in that it is normally limited to an intense expression among the SB keratinocytes where proliferation occurs (Bagnato et al., 1995), and may only be impacted by strong sensory stimuli that are sufficiently intense to cause a normal acute algesic response. Following PNT insults, ETA expression spread into superficial epidermal keratinocytes where it may drive a chronic algesic mechanism. Importantly, under normal conditions, human epidermal keratinocytes are replaced about every 4 weeks, however chronic NP patients seem to recapitulate the altered keratinocyte expression patterns. It is not yet known what mechanisms are involved in epidermal patterning after injury, or if such mechanisms are also altered in NP conditions (Albrecht and Rice, 2010, Hou et al., 2011). Ultimately, a better understanding of the mechanisms and precise time courses of cutaneous innervation and keratinocyte neural signaling pathologies in NP will enable the exploitation of topical therapeutic strategies (Peppin et al., 2015).

In summary, we show the important utility of the pig PNT model by demonstrating that pig skin structure and innervation is comparable to that of humans, and that PNT insult produces behavioral outcomes, decreased cutaneous innervation, and altered epidermal keratinocyte expression patterns that are consistent with behaviors and pathologies among human NP patients. Moreover, the pig provides a large animal model with similar physical stressors and metabolic properties of humans. The pig sciatic nerve PNT insult model provides copious skin surfaces amenable to longitudinal studies using skin biopsies to assess criteria for pre-treatment predictors and post-treatment outcomes, including the testing of topical compounded therapies. Therefore, the pig sciatic nerve PNT insult model has many properties indicating that it is an ideal translatable platform for the discovery, development, and pre-clinical safety and efficacy testing of novel therapeutic strategies for treating human NP.

## 5 Conflict of interest

All authors have nothing to disclose and there are no conflicts of interest present in the production of this research.
